# Time-dependent recruitment of GAF, ISGF3 and IRF1 complexes shapes IFNα and IFNγ-activated transcriptional responses and explains mechanistic and functional overlap

**DOI:** 10.1007/s00018-023-04830-8

**Published:** 2023-06-22

**Authors:** Agata Sekrecka, Katarzyna Kluzek, Michal Sekrecki, Mahdi Eskandarian Boroujeni, Sanaz Hassani, Shota Yamauchi, Kiyonao Sada, Joanna Wesoly, Hans A. R. Bluyssen

**Affiliations:** 1 Human Molecular Genetics Research Unit, Institute of Molecular Biology and Biotechnology, Faculty of Biology, Adam Mickiewicz University, Poznan, Poland; 2 High Throughput Technologies Laboratory, Faculty of Biology, Adam Mickiewicz University, Poznan, Poland; 3 Department of Genome Science and Microbiology, Faculty of Medical Sciences, University of Fukui, Fukui, Japan

**Keywords:** Immunology, Interferons, Jak/STAT signaling, GAF, ISGF3, IRF1, Integrative omics approach, Mechanism of transcriptional regulation

## Abstract

To understand in detail the transcriptional and functional overlap of IFN-I- and IFN-II-activated responses, we used an integrative RNAseq-ChIPseq approach in Huh7.5 cells and characterized the genome-wide role of pSTAT1, pSTAT2, IRF9 and IRF1 in time-dependent ISG expression. For the first time, our results provide detailed insight in the timely steps of IFNα- and IFNγ-induced transcription, in which pSTAT1- and pSTAT2-containing ISGF3 and GAF-like complexes and IRF1 are recruited to individual or combined ISRE and GAS composite sites in a phosphorylation- and time-dependent manner. Interestingly, composite genes displayed a more heterogeneous expression pattern, as compared to GAS (early) and ISRE genes (late), with the time- and phosphorylation-dependent recruitment of GAF, ISGF3 and IRF1 after IFNα stimulation and GAF and IRF1 after IFNγ. Moreover, functional composite genes shared features of GAS and ISRE genes through transcription factor co-binding to closely located sites, and were able to sustain IFN responsiveness in STAT1-, STAT2-, IRF9-, IRF1- and IRF9/IRF1-mutant Huh7.5 cells compared to Wt cells. Thus, the ISRE + GAS composite site acted as a molecular switch, depending on the timely available components and transcription factor complexes. Consequently, STAT1, STAT2 and IRF9 were identified as functional composite genes that are part of a positive feedback loop controlling long-term IFNα and IFNγ responses. More important, in the absence of any one of the components, the positive feedback regulation of the ISGF3 and GAF components appeared to be preserved. Together, these findings provide further insight in the existence of a novel ISRE + GAS composite-dependent intracellular amplifier circuit prolonging ISG expression and controlling cellular responsiveness to different types of IFNs and subsequent antiviral activity. It also offers an explanation for the existing molecular and functional overlap between IFN-I- and IFN-II-activated ISG expression.

**Supplementary Information:**

The online version contains supplementary material available at 10.1007/s00018-023-04830-8.

## Introduction

Interferons (IFN), initially discovered as antiviral proteins, can be divided into three subtypes IFN-I, IFN-II, and IFN-III. IFN-II or IFNγ, also named immune IFN, binds to the IFNγ receptor (IFNGR) and facilitates a more general immune response to pathogens. IFN-II is mainly produced by T lymphocytes and Natural Killer (NK) cells and modulates adaptive immunity as well as inflammation in response to foreign antigens or mitogens. Produced by many cell types, IFN-I is the main antiviral IFN subtype, mainly constituting IFNα and IFNβ subtypes. As part of a robust innate immune response, IFN-I collectively binds to the IFNα receptor (IFNAR) to potently inhibit viral infection. IFN-III consists of IFNλ1 to -4 subtypes, which largely are produced in a tissue-specific manner and bind the heterodimeric IFN-λ receptor (IFNLR) expressed only at certain cell types and tissues. These include macrophages, dendritic cells, plasmacytoid dendritic cells and respiratory epithelial cells. As such, IFN-λ confers more localized antiviral responses at the site of infection [1, 2].

Although IFN-I and IFN-II bind different cell-surface receptors expressed on most cell types, they both induce IFN-stimulated gene (ISG) expression through Janus kinase (JAK)-dependent phosphorylation of signal transducer and activator of transcription (STAT) 1 and STAT2. STAT1 homodimers, known as γ-activated factor (GAF), directly activate transcription of ISGs containing the IFN-II activation site [γ-activated sequence (GAS); consensus TTTCNNNGAAA] in response to both types of IFN. STAT1-STAT2 heterodimers together with interferon regulatory factor (IRF)9 (known as ISGF3) in response to IFNα expands the range of regulatory elements that can be targeted by the JAK-STAT pathway to the IFN-stimulated response element (ISRE; consensus AGTTTCN2TTTCN) [3, 4].

Although the core type I and type II IFN signaling pathways are distinct, their expression profiles show considerable overlap and are difficult to discern [5, 6]. Such overlap includes a similar behavior in phosphorylation of STAT1 and/or STAT2, expression of the ISGF3 and GAF components STAT1, STAT2 and IRF9 and IRF1 and expression of GAS and ISRE-containing genes in response to IFN-I and IFN-II. Over the years also a number of transcriptional mechanisms have been identified that could explain this overlap. For example, IFN-I-inducible STAT1-STAT2 heterodimers (GAF-like) can also directly bind a GAS element without IRF9 [7]. However, except for IRF1 only few other GAS-containing ISGs have been identified as direct targets of this type of heterodimers. Also, IRF1 has been shown to regulate transcription of ISGs in response to IFN-I and IFN-II. Thus, as a STAT1-target gene, IRF1 participates in secondary IFN-I and -II responses by activating transcription of ISRE-containing genes [8]. Likewise, non-canonical ISRE binding complexes, including STAT1/IRF9 (homodimer of STAT1 complexed with IRF9) and ISGF3^II^ (heterodimer of pSTAT1 and unphosphorylated STAT2 together with IRF9), have been identified to regulate IFNγ-activated transcription of ISRE genes [4, 6, 9]. Finally, mechanistic overlap between both types of IFN can also be explained by the identification of ISGs that contain both ISRE and GAS elements and thus can be activated by both type I and II IFNs [6, 10–12], through potential mechanisms of co-binding of ISGF3, IRF1, or GAF complexes [5, 11, 13, 14].

The general paradigm of IFN-I and or IFN-II signaling displays a robust and transient phosphorylation pattern of STAT1 and STAT2 followed by a similar ISG expression profile that decreases over time. Recently, more complexity has been identified in IFN signaling with more sustained ISG expression patterns [5, 15–18]. This prolonged response was shown to rely on sustained expression of the components of ISGF3 and GAF (STAT1, STAT2 and IRF9) and IRF1 as part of a positive feedback loop. Additionally, a novel role of unphosphorylated ISGF3 (U-ISGF3), U-GAF and IRF1 has been proposed in basal as well as long-term ISG expression [5]. However, the exact timely steps that take place during IFN-activated feedback regulation and how it controls ISG transcription and long-term cellular responsiveness to IFN-I and IFN-II is currently not clear.

Using a genome-wide RNAseq-ChIPseq integrative approach, our results identify a novel class of IFN-I and IFN-II responsive genes that use ISRE and GAS composite sites to recruit STAT1 and STAT2-containing ISGF3 and GAF-like complexes and IRF1 for optimal expression. Moreover, they support the existence of an analogous transcriptional regulatory mechanism of IFNα and IFNγ inducible genes, in which STAT1- and STAT2-containing ISGF3 and GAF-like complexes and IRF1 are recruited to individual or combined ISRE and GAS composite sites in an IFN- and time-dependent manner. In addition, we present proof for the involvement of an ISRE and GAS composite-dependent regulatory system in IFN-activated positive feedback regulation of the STAT1, STAT2, IRF9 and IRF1 genes and long-term response to IFNs, which depends on phosphorylation and expression of ISGF3, IRF1 and GAF-like components. These findings provide further insight in the existence of a novel ISRE + GAS composite-dependent intracellular amplifier circuit that offer an explanation for the existing molecular and functional overlap between IFN-I and IFN-II activated ISG expression.

## Materials and methods

### Cell lines

The human hepatocellular carcinoma cell lines—Huh7.5 WT, Huh7.5 IRF9KO, Huh7.5 STAT1KO, Huh7.5 STAT2-KO—were described earlier [17]. Huh7.5 IRF1KO and Huh7.5 IRF9/IRF1 dKO cell lines were generated using a custom-designed plasmid pZG22D03-2 (ZGene Biotech Inc.) in combination with the CRISPR-Cas9 system [19]. Briefly, Huh7.5 or Huh7.5 IRF9KO cells were transfected with the Cas9D10A nickase/EGFP vector pZG22D03-2 that carries sgRNAs (g1—GCTTGGCAGCATGCTTCCATGGG; g2—ATGCCTGTTTGTTCCGGAGCTGG) targeting exon 3 of human *IRF1* (NM_002198.3). After transfection for 24 h, EGFP-positive cells were sorted for single cells into 96-well plates. The clones were picked and screened for indel formation and western blotting for protein expression 2 weeks later.

### Cell culture + treatment

Huh7.5 WT and KO cell lines were maintained in DMEM (11, IITD PAN Wrocław) supplemented with 10% FBS (10500-064, Thermo Fisher Scientific (TFS)), 1% MEM NEAA (11140-035, TFS), 2 mM l-glutamine (X0550, BioWest), 100 IU/ml penicillin, 100 µg/ml streptomycin and 250 ng/ml amphotericin B (A5955, Sigma-Aldrich): full cell culture medium. For IFN treatment, cells were seeded in full cell culture medium, which was replaced the next day with starving medium (DMEM, 1% FBS, 1% MEM NEAA, 2 mM l-glutamine, 100 units/ml penicillin, 0.10 mg/ml streptomycin and 0.25 μg/ml amphotericin B) for 8 h before starting the treatment. IFNα (1000 U/ml, IF007, MERCK) or IFNγ (10 ng/ml, IF002, MERCK) was added for indicated time-points.

### Western blot

Protein isolation, protein quantification and immunoblotting were carried out as described previously [20]. Primary antibodies included: tSTAT1 (CST, 14994, D1K9Y) 1:1000, pSTAT1 (CST,7649, D4A7) 1:400, tSTAT2 (CST, 72604, D9J7L) 1:500, pSTAT2 (CST, 88410, D3P2P) 1:500, IRF1 (CST, 8478, D5E4) 1:300, IRF9 (SantaCruz, sc-365893, H-10) 1:500, α-Tubulin (abcam, AB52866, EP13332Y; SantaCruz, sc-398103, A-6) 1:2000; 1:500. Secondary HRP-conjugated antibodies included: Rabbit ab (Sigma-Aldrich, A9169) 1:20.000, Mouse ab (Sigma-Aldrich, A9044) 1:20.000, Mouse ab (SantaCruz, sc-2954) 1:5000.

### RNA isolation and quantitative reverse transcription-PCR (qRT-PCR) analysis

Total RNA was isolated using TRI-REAGENT (TRI118, MRC) followed by a column-based Total RNA Zol-Out™ D kit (043, A&A Biotechnology) following the manufacturer’s protocol.

500 ng of total RNA was subjected to reverse transcription according to the protocol of the RevertAid First-Strand Synthesis Kit (K1622, TFS). Transcript quantification was performed by qPCR with Maxima SYBR Green/ROX qPCR Master Mix (K0223, TFS) on the CFX Connect Thermal Cycler System (Bio-Rad). Target gene levels were normalized to the housekeeping gene—glyceraldehyde-3-phosphate dehydrogenase (GAPDH). Data were transformed as described previously [21]. Forward and reverse primers used in experiments are depicted in Table S1. If not indicated differently, results are presented as mean ± SEM for two independent biological repeats. Graphs are prepared using GraphPad Prism 8.3.0.

### RNA-seq library preparation and sequencing

RNA was isolated from Huh7.5 WT and KO cells exposed to IFNα or IFNγ for 0, 2, 4, 8, 24, 48, 72 h. RNA was quantified using Qubit RNA BR (Broad Range) assay kit (Q10210, TFS) and quality was assessed using Experion™ RNA StdSens Analysis Kit (700-7103, Bio-Rad) according to the manufacturer’s protocol. Only RNA with RNA Integrity Number (RIN) higher than 9 was considered for library preparation. RNA-seq libraries were prepared in two biological replicates from 1ug of total RNA using NEBNext^®^ Ultra™ II RNA Library Prep Kit for Illumina^®^ (E7770, NEB) together with NEBNext Poly(A) mRNA Magnetic Isolation Module (E7490, NEB) and NEBNext^®^ Multiplex Oligos for Illumina^®^ (E7335, NEB) according to manufacturer’s protocol. Libraries were quantified using Qubit dsDNA HS assay kit (Q32851, TFS) and quality and fragment distribution were examined with Agilent High Sensitivity DNA kit (5067-4626, Agilent Technologies). Sequencing was performed on the NextSeq500 (HighOutput SR75) in Lexogen, BioCenter in Vienna, Austria.

### RNA-seq data analysis

Fastq files were aligned using STAR version 2.7.3a against the Homo_sapiens.GRCh38.dna.primary_assembly genome build (release-100) [22]. Quality control assessments were made using FastQC and reports combined with MultiQC [23, 24]. Gene counts (reads aligned to each gene of each sample) were generated using FeatureCounts v1.6.2 with default parameters [25]. Genes with low counts (below 10 in all time-points) were considered “non-expressed” and filtered out for the downstream testing.

To determine differentially expressed genes (DEG), counts normalization and DEG analysis were performed using the DESeq2 v.1.30.1 package [26] in R version 4.0.3 [27]. The likelihood ratio test (LRT) was used to identify genes that respond to IFN treatment over time. False discovery rate (FDR)-adjusted *q*-values (5% threshold) were calculated by Benjamini–Hochberg procedure. The log2(fold change) FC also was calculated for each gene. Genes with adjusted *p*-values (*p*_adj_) less than 0.05 and log2FC > 0.5 were considered as DEGs.

### Expression cluster generation

The degPatterns function from DEGreport package v1.30.0 was used to determine sets of genes that exhibit similar expression patterns across sample groups [28].

### Heatmap generation

Heatmaps visualizing transcriptional response to IFNα and IFNγ treatment were created using pheatmap v1.0.12 and ComplexHeatmap v2.10.0 packages [29, 30]. For selected genes, normalized counts obtained from DESeq2 were extracted and subjected to hierarchical clustering (only by row) with default clustering method: complete, Euclidean distance. For plotting, row scaling with *Z*-scores was performed. Color scale indicates the expression change over time for each sample compared to the expression of the non-treated control. Colors represent high (red) and low (blue) normalized intensity, respectively.

### Identifying and visualizing enriched GO terms

Lists containing gene names were subjected to Gene Ontology terms enrichment analysis using The Database for Annotation, Visualization and Integrated Discovery (DAVID) v6.8 [31, 32]. Only terms with a calculated False Discovery Rate (FDR) < 0.05 were considered statistically significant.

### Chromatin immunoprecipitation (ChIP)-seq

Chromatin was quantified using Qubit dsDNA HS (High Sensitivity) assay kit and Qubit™ 3 Fluorometer (Invitrogen). Two biological replicates were prepared for sequencing. For library preparation, NEBNext^®^ ChIP-Seq Library Prep Master Mix Set for Illumina^®^ (E6240, NEB) and NEBNext^®^ Multiplex Oligos for Illumina^®^ were used according to the manufacturer’s protocol. Libraries were quantified using Qubit dsDNA HS assay kit and quality and fragment distribution were examined with Agilent High Sensitivity DNA kit. Sequencing was performed on the NextSeq500 (HighOutput SR75) in Lexogen, BioCenter in Vienna, Austria.

### ChIP-seq data analysis

All ChIP-seq experiments were performed in duplicate and were scored against input DNA. After quality assessment of raw reads with FastQC [24], ChIPseq data was analyzed according to ENCODE Transcription Factor and Histone ChIP-Seq processing pipeline v3 (https://github.com/ENCODE-DCC/chip-seq-pipeline2) with default parameters (ENCODE Project Consortium, 2012).

Briefly, sequencing reads were aligned to hg38 v29 genome (https://www.encodeproject.org/files/ENCFF110VAV/) using Bowtie2 v2.4.1 [33] with mapping quality threshold 30. Subsequently, duplicates were marked using Picard Tools (http://broadinstitute.github.io/picard/). Peaks were called with SPP, FDR threshold set to 0.01. Afterwards, Irreproducible Discovery Rate (IDR) was used to determine an optimal number of reproducible peaks between biological replicates. An IDR score threshold of 0.02 was used to obtain optimal set of peaks. All peak sets were then screened against a specially curated blacklist of regions in the human genome provided within ENCODE pipeline and peaks overlapping the blacklisted regions were discarded. *p*-value signal tracks for each replicate and replicates pooled were generated with MACS2.

All samples passed quality check based on thresholds set by ENCODE, detailed description of all parameters can be found at: https://www.encodeproject.org/data-standards/terms/#concordance. To obtain the high-quality data, we discarded peaks with the lowest score from the peak list using a cut-off of 10–20% of the maximum peak score of a specific antibody. As a result, we obtained ~ 4000 top-scored peaks for each antibody, except IRF9. Because of the low quality of IRF9 ChIP we used the 5% threshold and still obtained only 1360 unique peaks.

### Deposited sequencing data

RNA sequencing and ChIP sequencing data are accessible at NCBI GEO DataSets under accession number: SuperSeries GSE222668.

### Visualization in the Integrative Genomics Viewer

To visualize the ChIP-seq in the Integrative Genomics Viewer (IGV) [34], BAM files were prepared using Bowtie2 aligner v2.4.1 [33] and converted using bamCoverage (deepTools2 v3.5.0) [35] to bigwig format. Snapshots were taken to present them in figures.

### Binding profiles

To generate pSTAT1 and/or pSTAT2 binding profiles upon IFNα and IFNγ treatment at early time-points, the matrices that represent bigWig signal over selected genomic intervals have been quantified.

For each treatment, *p*-value signal tracks from pooled replicates for selected time-points and bed file with peaks annotated to promoters of integrated genes were used. Read numbers were computed across 4 kb region centered on ChIP-Seq peak summits with computeMatrix function from deepTools v3.5.0 (reference-point TSS, upstream region 3000 bp, downstream region 1000 bp) [35]. Further steps were performed with profileplyr_1.10.0 package [36]. First, k-means clustering of the signal across the genomic intervals of gene promoters was performed with pheatmap package [30]. Mean range signal for each cluster was subsequently visualized as boxplots with ggplot2 [37].

### Binding site motifs identification

A combined list of all unique peaks from all samples was prepared with custom R script and used to identify enriched transcription-factor-binding motifs by Hypergeometric Optimization of Motif EnRichment (HOMER) v. 4.9.1 [38] and select GAS and ISRE motifs from HOMER database (Figs. S2 and S3). To refine motif detection, sites recognized by each matrix were counted and intersecting sets were visualized using UpSet plots generated by the UpSetR tool [39]. The final set of selected matrices for binding elements annotation (4 for GAS and 3 for ISRE) are shown in Figs. S2B and S3B. Motif logos were generated using universalmotif package v1.12.1 [40].

### Data integration using BETA

Based on the binding profiles for pSTAT1, pSTAT2, IRF1 and IRF9, TF-specific gene lists were prepared. Next, BETA 1.0.7 [41] was implemented for the integration analysis using up-regulated, adjusted *p*-value (*p*_adj_ < 0.05) genes from RNA-seq and the peaks from ChIP-seq, followed by extraction of up-regulated integrative genes from all time-points (Fig. S4). Then, the integrative gene list was refined by the identification of overlapping genes between the integrative list and the gene lists from the binding profile.

The IFNα- and IFNγ-specific networks from the refined integrative list were constructed and visualized using Cytoscape v3.8.2 [42] and ggplot2 v3.3.5 [37]. In addition the ClueGo v2.5.8 Cytoscype plug-in [43] was used to overlay the functional terms and visualize the enriched GO terms. Criterium for inclusion (for the list genes > 200) was the 3–8 GO tree interval, a minimum of three genes from the uploaded list found to be associated to a term, and that these genes represent at least 4% of the total number of associated genes.

### Site-directed mutagenesis in combination with promoter-luciferase expression analysis

Genomic DNA from Huh7.5 cells was isolated using Genomic MINI kit (116, A&A Biotechnology) according to the manufacturer’s protocol, including the optional RNAse digestion step (EN0531, TFS) and used as a template for amplifying the pre-selected gene promoter sequences (Key Resources Table). All experiments were designed using Geneious Prime software (https://pubmed.ncbi.nlm.nih.gov/22543367/). The pXPG vector was used as a backbone for all luciferase constructs (Plasmid #71248, Addgene). Plasmid DNA was linearized using SmaI (R0141S, NEB). All cloning procedures were according to the Gibson assembly approach, with the usage of NEBuilder^®^ HiFi DNA Assembly (E2621, NEB) and specifically designed primers having the 15nt overhangs, complementary to SmaI cutting site (Key Resources Table). Assembling the plasmid and DNA fragments was performed at 50 °C for 45 min with NEBuilder HiFi DNA Assembly Master Mix (E2621, NEB). Purified plasmids were isolated using column-based Monarch^®^ Plasmid Miniprep Kit (T1010L, NEB) followed by Sanger sequencing (Molecular Biology Techniques Laboratory at Adam Mickiewicz University in Poznan, Poland). Obtained sequences were aligned using the BLAT tool available UCSC Genome Browser.

For luciferase assays, 10 × 10^3^ of the Huh7.5 cells were seeded into 96-well plates. For analyzing the ISG promoter activity, the 70 ng of investigated plasmid were transfected using Lipofectamine3000. Simultaneously, as a reference, 30 ng of pRL Renilla Luciferase Control Reporter Vector (pRL-SV40; [E2231, Promega]) was co-transfected to the cells. After 24 h of incubation, IFNα (1000 U/ml) or IFNγ (10 ng/ml) was added for 8 h treatment. Luciferase activity was measured using Dual-Glo^®^ Luciferase Assay System (E2920, Promega) according to the manufacturer’s protocol. The luminescence level was detected and measured with THE SPARK^®^ multimode microplate reader (TECAN). Each experiment was conducted in four technical and two biological replicates. Relative Luciferase Units (RLU) were calculated as the Firefly luciferase activity level normalized using Renilla luciferase activity level from the same sample. Graphs are prepared using GraphPad Prism 8.3.0.

To generate GAS and ISRE sequence mutations in pre-selected gene promoters, site-directed mutagenesis was performed using NEBuilder^®^ HiFi DNA Assembly Master Mix according to manufacturer’s protocol. Cloning, plasmid isolation and sequencing was as above. Flanking primers with mutations are listed in the Key Resources Table. For the generation of double-mutant plasmids, the single-mutant plasmids were used as a template.

## Results

### Characterization of time-dependent IFNα and IFNγ responses

**Fig. 1:**
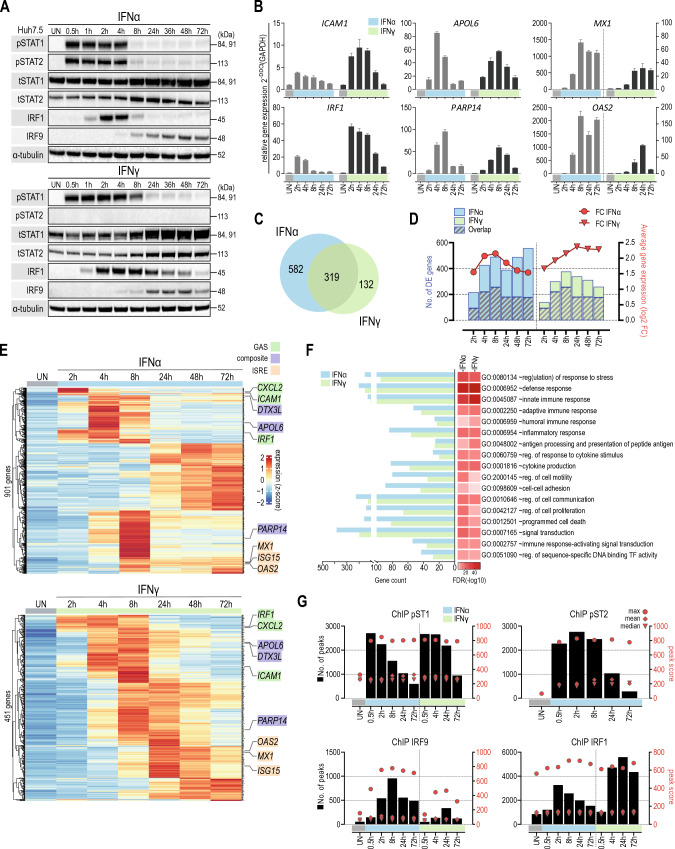
GAS, ISRE and composite genes are commonly involved in IFNα- and IFNγ-mediated transcriptional responses and chromatin interactions. **A** Huh7.5 cells were treated with IFNα (1000 U/ml) or IFNγ (10 ng/ml) for the indicated times. The expression levels of STAT1, STAT2, IRF9 and IRF1 were evaluated by immunoblotting; *p* phosphorylated, *t* total. **B** Expression profile of pre-selected ISGs quantified using qPCR. Cells were left untreated or stimulated with IFNα (1000 U/ml) or IFNγ (10 ng/ml) for the indicated time. Relative expression (over GAPDH) was estimated. *n* = 3; mean ± SEM. **C** Venn diagram based on RNA-seq results showing total numbers of uniquely and commonly up-regulated genes in Huh7.5 cells after IFNα or IFNγ stimulation (log2FC > 0.5; *p*_adj_ < 0.05). **D** Number of up-regulated genes (log2 FC > 0.5 and *p*_adj_ < 0.05) in each time-point is indicated using color bars (corresponding values—left *y*-axis). The area of the bar covered with lines represents the number of overlapping genes between analogous time-points for both IFNs. Average gene expression is shown using red lines with symbols (corresponding values—right *y*-axis). **E** Heatmaps generated from the expression values of all up-regulated genes in Huh7.5 cell treated with IFNα or IFNγ. Each row represents one gene. Counts were normalized using *Z*-score. Pre-selected genes are marked on each graph. The color of the gene name corresponds to the binding site recognized in the promoter of the gene. **F** Gene Ontology terms enrichment analysis of up-regulated genes by IFNα or IFNγ (significant enrichment considered with FDR < 0.05). **G** Characterization of ChIP-seq peak score and distribution for pSTAT1, pSTAT2, IRF9 and IRF1 in IFNα- and IFNγ-stimulated Huh7.5 cells. Black bars represent a number of peaks called using spp and thresholded using Irreproducible Discovery Rate (IDR) for each time-point separately (corresponding values—left *y* axis). Red circles, triangles and rhombi represent the maximum, median and mean peak values, respectively (corresponding values—right *y* axis)

To obtain further insight into the overlapping IFN-I- and IFN-II-activated transcriptional responses in Huh7.5 WT cells, we first characterized the IFNα- and IFNγ-induced expression and phosphorylation of ISGF3 and GAF components, i.e., STAT1, STAT2 and IRF9 and IRF1 (Fig. 1A). IFNα treatment for different time-points (0, 0.5, 1, 2, 4, 8, 24, 36, 48, 72 h) resulted in a transient increase in phosphorylation of STAT1 and STAT2 between 0.5 and 4 h, after which it rapidly diminished to lower but still detectable levels even after 72 h. IRF1 expression clearly correlated with the transient phosphorylation pattern of STAT1 and STAT2, with a maximum expression at 2 h followed by a decrease in time. In contrast, expression of STAT1 and STAT2, as well as IRF9, exhibited a prolonged character and increased in expression until 72 h after treatment. Compared to IFNα, time-dependent IFNγ treatment resulted in a similar, but more prolonged behavior of STAT1 phosphorylation, but not STAT2, and accumulation of IRF1, STAT1, STAT2 and IRF9 (Fig. 1A).

This marks the positive feedback regulation of the ISGF3 and GAF components observed in response to IFNα as well as IFNγ.

The known GAF-target and GAS-containing genes ICAM1 and IRF1 showed an early and transient response to both types of interferon (Fig. 1B), with a stronger and more delayed effect upon IFNγ stimulation. This correlated with the phosphorylation pattern of STAT1 and/or STAT2 depending on the type of IFN (Fig. 1A). The expression of the known ISGF3 and IRF1-target and ISRE-containing genes MX1 and OAS2, on the other hand, followed a more delayed and prolonged pattern, with a much stronger effect upon IFNα treatment (Fig. 1B). This followed the presence of phosphorylated STAT1 and/or STAT2 in combination with the increased expression of IRF9 and IRF1 induced by the different IFNs (Fig. 1A). The Composite (GAS + ISRE-containing) genes APOL6 and DTX3L, on the other hand, exhibited a more intermediate response to both types of interferon, with a comparable efficacy (Fig. 1B). This could point to the combined involvement of both GAS and ISRE sites with GAF, ISGF3 and IRF1 complexes, depending on time and type of IFN.

### GAS, ISRE and composite genes are commonly involved in IFNα and IFNγ mediated transcriptional responses

To obtain detailed insight into the time-dependent IFN-I- and IFN-II-activated transcriptional responses and chromatin interactions, we first performed RNAseq on RNA isolated from Huh7.5 WT cells treated with IFNα or IFNγ for 0, 2, 4, 8, 24, 48 and72 h. Using differential expression analysis (DESeq) we identified 901 IFNα- and 451 IFNγ-induced genes (Fig. 1C), of which 319 genes that were commonly up-regulated (Fig. 1C). In general, the potency of transcriptional responses to IFNα was higher in comparison to IFNγ, whereas a more transient expression profile could be observed for IFNα up-regulated genes over time (Fig. 1D).

Interestingly, heatmaps presenting the expression pattern of IFNα and IFNγ up-regulated genes in time, identified different clusters and further illustrated important overlap between transcriptional responses to both types of IFN. Closer inspection of a pre-selection of these different types of ISGs identified analogical clusters in IFNα- and IFNγ-dependent heatmaps (Fig. 1E). Moreover, it confirmed the early and transient nature of the expression of GAS-containing genes (IRF1, ICAM1, CXCL2) as compared to the more delayed and prolonged pattern of ISRE-containing genes (ISG15, MX1, OAS2) in response to both types of IFN. The composite (GAS + ISRE-containing) genes APOL6 and DTX3L, PARP14, on the other hand, exhibited a more intermediate as well as transient response to both types of IFN (Fig. 1E). Moreover, GO analysis of IFNα and IFNγ up-regulated genes revealed significant enrichment in biological terms connected to, innate, adaptive and humoral immune response, response to stress, cytokine production or immune response-activating signal transduction. They all reflect antiviral and pro-inflammatory biological functions and also pointed to the functional overlap between IFNα and IFNγ responses (Fig. 1F).

Next, we characterized the genome-wide binding of the GAF and ISGF3 components and IRF1 to the regulatory regions of IFNα and IFNγ up-regulated genes. Thus, we performed ChIP-seq on chromatin from Huh7.5 cells exposed to IFNα (0, 0.5, 2, 8, 24 and 72 h; pSTAT1, pSTAT2, IRF9 and IRF1) and IFNγ (0, 0.5, 4, 24 and 72 h; pSTAT1, IRF9 and IRF1). Because of significant lower ChIP quality results with the IRF9 antibody, the number of identified IRF9 peaks was noticeably lower than compared to other antibodies and also associated with lower scores. Under these conditions, the peak score distribution followed a transient pattern for pSTAT1, pSTAT2, IRF1 and IRF9 in response to IFNα, being the highest for pSTAT1, pSTAT2 and IRF1 at 2 h and for IRF9 at 8 h (Fig. 1G). Likewise, exposure to IFNγ increased the genome-wide number of pSTAT1, IRF9 and IRF1 binding peaks/sites, however with a more prolonged character. pSTAT1 displayed the strongest signal at 4 h, whereas peak scores for IRF9 and IRF1 were maximum at 24 h. Opposite to IRF9, which peak score values were clearly lower than after IFNα stimulation, a higher number of annotated peaks for IRF1 could be observed in comparison to IFNα (Fig. 1G). In line with the lack of STAT2 phosphorylation after IFNγ treatment (Fig. 1A), no genome-wide binding of STAT2 could be detected under these conditions (not shown). Although a more transient binding pattern was detected for pSTAT1 and/or pSTAT2, IRF1 and IRF9 in response to IFNα as compared to IFNγ, recruitment was still clearly detectable after 72 h (Fig. 1G). Strikingly, only for IRF1, high peak scores could already be identified in the absence of IFN (Fig. 1G).

Further analysis indicated that the pSTAT1, pSTAT2, IRF9 and IRF1 binding peaks were located predominantly in introns and promoters of IFNα- and IFNγ-responsive genes (Fig S1A, B). These regions corresponded to the presence of individual GAS sites, commonly bound by pSTAT1 and pSTAT2 in response to IFNα, and pSTAT1 after IFNγ treatment, or ISRE sites, recruiting pSTAT1, pSTAT2, IRF9 and IRF1 after IFNα and pSTAT1, IRF9 and IRF1 after IFNγ treatment (Figs. S1C, D, S2 and S3). In addition, binding regions also contained GAS + ISRE composite sites, which showed similar binding characteristics as ISRE genes (Fig. S1C, D). This offers clear evidence for the existence of a GAS + ISRE composite gene group, which together with GAS and ISRE genes are commonly involved in IFNα and IFNγ mediated transcriptional responses.

### Time-dependent IFNα and IFNγ transcriptional responses depend on differential binding of GAF, ISGF3 and IRF1 complexes to GAS, ISRE and composite genes

Subsequently, we performed an integrative analysis of our RNAseq-ChIPseq data. By concentrating on the promoter/5′UTR regions with annotated GAS and/or ISRE motifs, our multi-omics data integration identified 319 IFNα-responsive genes that bound pSTAT1, pSTAT2, IRF9 and/or IRF1 at least at one time-point. Likewise, 286 IFNγ-inducible genes were selected that bound pSTAT1, IRF9 and/or IRF1 (Fig. S4).

**Fig. 2:**
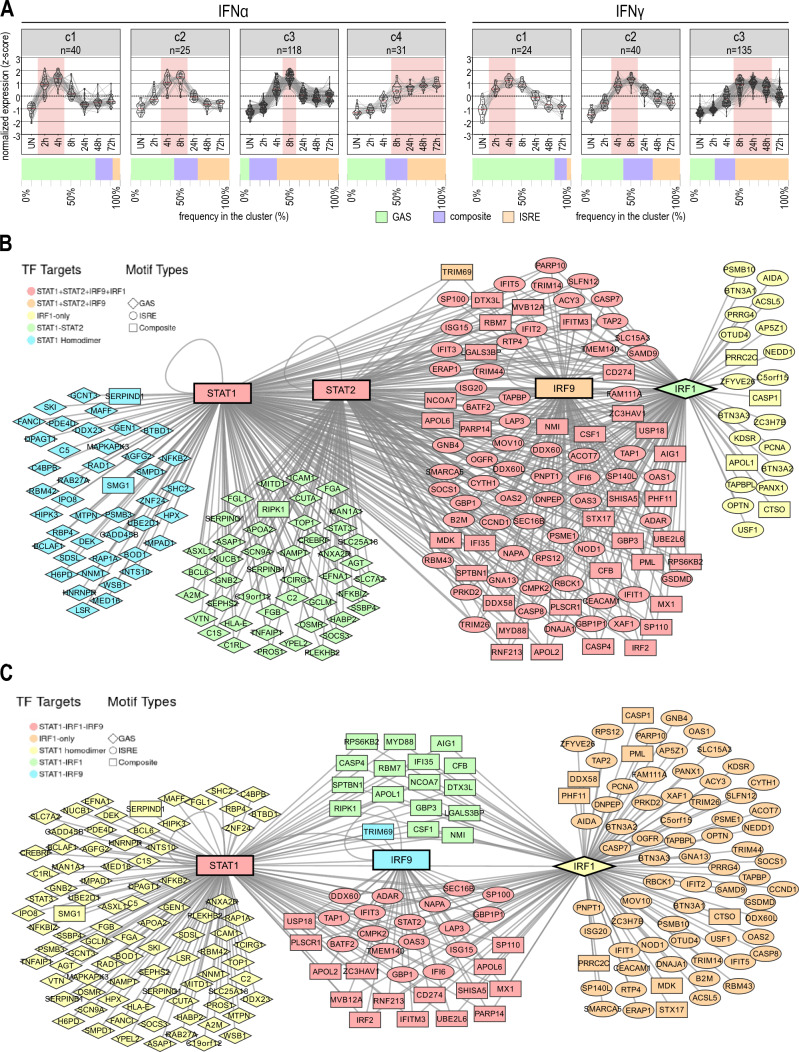
Time-dependent transcriptional overlap of GAS, ISRE and composite genes is mediated by differential binding of GAF, ISGF3 and IRF1 complexes. **A** Clustering of integrated genes based on RNA-seq results. Each gray line connecting dots between time-points represents a single gene. Expression was normalized using *Z*-score. The lines within each violin plot represent the median (red) and first and third quartiles (black); red area indicates time-points with the highest expression in each cluster; *n* = number of integrated genes in the cluster. Bar chart below each cluster represents the frequency of integrated gene promoters with GAS, ISRE or both sites annotated. **B** Network of integrated genes in IFNα- and IFNγ-stimulated Huh7.5 cells. Each node represents one gene. The shape of the node indicates the regulatory element present in the gene promoter (rhomb—GAS; square—ISRE; oval—composite). As central nodes STAT1, STAT2, IRF9 and IRF1 are placed, which are at the same time the symbols of the binding components and ISGs. Each line symbolizes the binding of one of the four components. Genes are color coded to indicate the signaling proteins bound to the gene promoter. The network was created using Cytoscape and visualized using ggplot

Comparing their expression profiles, several important clusters could be recognized in response to IFNα and IFNγ that distinguished early (maximum 2–4 h), intermediate (max. 4–8 h) and late (max. > 8 h) profiles (Fig. 2A). Interestingly, for both IFNα and IFNγ responses GAS-containing genes were predominantly present in the early cluster, either as a solitary element or as GAS + ISRE composite site. In contrast, ISRE-containing genes formed the majority in the intermediate and late clusters, mostly as single motif sites or as composite sites (Fig. 2A). This pointed to a mechanistic overlap and correlated with an early role of GAS-targeting GAF complexes and the later importance of ISGF3 and/or IRF1.

Comparative analysis accordingly identified 108 IFNα- and 75 IFNγ-specific integrated genes (Fig. S4). Detailed characterization of these specific genes unraveled GAS, ISRE and composite genes (Table S2). Interestingly, IFNα-specific genes consisted of a similar distribution of ISRE (40), composite (31) and GAS (37) genes, whereas IFNγ-specific genes were mainly GAS-containing (GAS: 49; composite genes: 9; ISRE: 17). According to GO analysis, the main function of these IFNα- and IFNγ-specific genes were connected to the defence response and immune system process, and in case of IFNγ-specific genes also to RNA biosynthetic process (data not shown).

On the other hand, the integrative analysis uncovered 211 IFNα and IFNγ commonly integrated genes. These commonly integrated genes included 86 GAS, 82 ISRE and 43 composite genes (Fig. S4; Table S3). Comparing the proximal promoters of these 43 common composite genes, uncovered a random organization [ISRE-GAS (+) or GAS-ISRE (−)], with a distance varying between 0 and < 415 bp (Table S4). Analyzing recruitment of pSTAT1, pSTAT2, IRF9 and/or IRF1 to these 211 common genes revealed different binding characteristics (Fig. 2B, C). For example, GAS genes bound pSTAT1 alone or pSTAT1 together with pSTAT2 in response to IFNα, which coincided with individual or combined roles of GAF and GAF-like complexes (Fig. 2B). Upon IFNγ treatment only pSTAT1 was recruited, which pointed to the sole role of GAF (Fig. 2C). In contrast, binding characteristics to ISRE genes in response to IFNα, involved collective recruitment of all ISGF3 components (pSTAT1, pSTAT2, IRF9) with or without IRF1, or only IRF1 (Fig. 2B). IFNγ predominantly directed IRF1 binding to these genes, but less frequently also IRF9 and pSTAT1 (Fig. 2C). Interestingly, Composite genes exhibited combined features of GAS and ISRE-containing genes, with the collective recruitment of pSTAT1, pSTAT2, IRF9 and IRF1 (and less frequently only ISGF3 components or only IRF1) after IFNα stimulation (Fig. 2B) and pSTAT1 + IRF1 and less frequently IRF9 after IFNγ (Fig. 2C). This could point to the involvement of GAF and/or GAF-like complexes in collaboration with ISGF3 and/or IRF1.

**Fig. 3:**
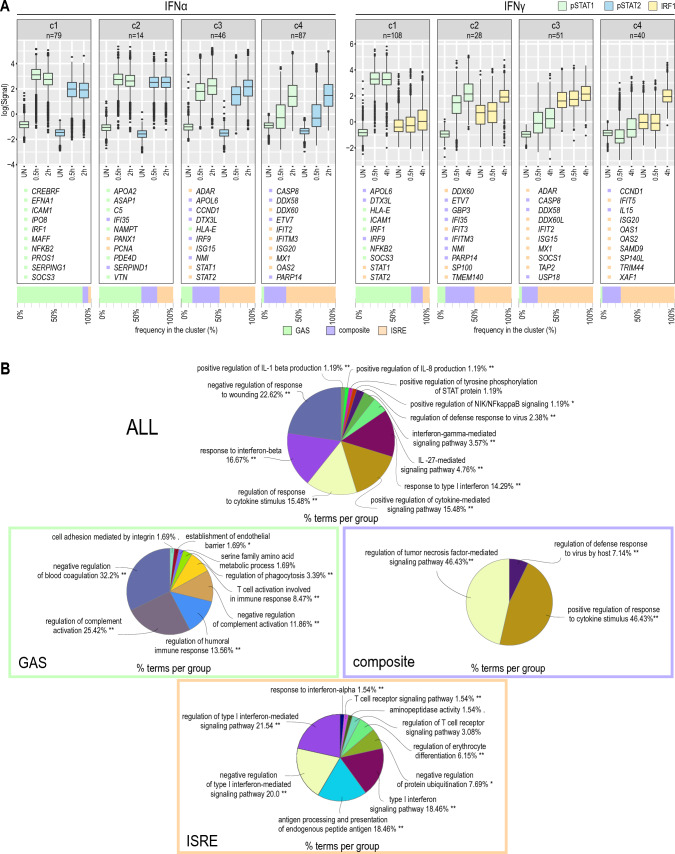
Differential binding of GAF, ISGF3 and IRF1 complexes to IFNα- and IFNγ-activated GAS, ISRE and composite genes mediates mechanistic and functional overlap. **A** Gene clustering based on ChIP-seq results. Boxplot represents the normalized scores of pSTAT1, pSTAT2 or IRF1 annotated peaks. Scores were obtained after IDR and only top-scored peaks were used in the analysis (see “Materials and methods”); *n* = number of integrated genes in cluster. The representative integrated genes are listed for each cluster with color referring to the binding site recognized in the gene promoter; *p* phosphorylated. Bar chart below each cluster represents the frequency of integrated gene promoters with GAS, ISRE or both sites annotated. **B** Gene ontology analysis for all commonly integrated genes for IFNα- and IFNγ-stimulated Huh7.5 cells or after dividing them into groups based on binding sites annotated in the gene promoters. Analysis performed using ClueGO tool for Cytoscape; *p < 0.05; **p < 0.01; ***p < 0.001

We also performed a cluster analysis of commonly IFNα- and IFNγ-integrated genes based on their time-dependent recruitment of pSTAT1 and pSTAT2 upon IFNα treatment and pSTAT1 and IRF1 after IFNγ treatment (Fig. 3A). Interestingly, the binding profile of GAS genes in response to both IFNα and IFNγ displayed a clear early character, corresponding with early expression. In comparison, the binding profile of composite and ISRE genes was delayed and prolonged, which correlated with a more intermediate and later expression pattern to both types of IFN (Fig. 3A).

Finally, enrichment analysis of these groups revealed significant enrichment in biological terms connected to defense response to virus and IFN and cytokine signaling (Fig. 3B). Particularly, GAS genes reflected more specific biological terms connected to complement activation and blood coagulation or establishment of endothelial barrier, whereas composite genes were explicitly linked to tumor necrosis factor-mediated signaling or further positive amplifying the cytokine-dependent signal. Finally, ISRE genes were specifically associated with antigen processing and presentation, T cell receptor signaling and IFN-I signaling.

### STAT1, STAT2 and IRF9 control onset and progression of IFN responses

To obtain further insight into the role of STAT1, STAT2, IRF9 and IRF1 in IFN-I- and IFN-II-activated transcriptional responses, we generated STAT1-, STAT2-, IRF9-, IRF1- and IRF9/IRF1-mutant Huh7.5 cells (Fig. S5). As compared to wt cells (Fig. S5A), knocking out STAT1 dramatically delayed and prolonged the phosphorylation pattern of STAT2 in response to IFNα, which correlated with the prolonged expression pattern of STAT2, IRF1 and IRF9 (Fig. S5B). The response to IFNγ in these cells was completely abrogated, as marked by the absence of phosphorylated STAT2 and expression of IRF1 and IRF9 (Fig. S5B). Knocking out STAT2 resulted in a significant decrease in STAT1 phosphorylation levels, displaying a bi-phasic character in response to IFNα and a clear transient pattern after IFNγ treatment. IRF1 expression clearly correlated with this phosphorylation pattern of STAT1, with a more prolonged expression after IFNα treatment as compared to early and transient in response to IFNγ. In contrast, the expression of STAT1 and IRF9 under these conditions exhibited a prolonged character and increased even after long-term treatment, similar to wt cells (Fig. S5C). Knocking out IRF9 predominantly effected the IFNα response, with a weaker and more transient phosphorylation pattern of STAT1 and STAT2 slowly decreasing over time until 72 h. IRF1 expression clearly correlated with this phosphorylation pattern of STAT1 and STAT2, being transient but more prolonged as compared to wt cells. In contrast, the expression of STAT1 and STAT2 under these conditions exhibited a prolonged character and increased even after long-term treatment, similar to wt cells. The response to IFNγ in these cells was similar to wt cells, displaying a transient phosphorylation of STAT1 reflecting the increase in IRF1 expression. Expression of STAT1 and STAT2 exhibited a prolonged character and increased even after long-term treatment (Fig. S5D). IRF1KO cells responded opposite to IRF9KO cells, with a transient phosphorylation pattern for STAT1 and STAT2 after IFNα treatment (that mirrored wt cells), and prolonged phosphorylation of STAT1 after IFNγ stimulation. In these cells, both types of IFN induced a similar expression pattern of STAT1, STAT2 and IRF9, however being more prolonged as observed in IRF9KO cells (Fig. S5D). Finally, in IRF1/IRF9 double KO cells, phosphorylation of STAT1 and STAT2 in response to IFNα and STAT1 in response to IFNγ exhibited an extremely prolonged pattern. More important, in IRF1/IRF9 double KO cells, the IFNα- and IFNγ-mediated increase in STAT1 and STAT2 proteins was still observed (Fig. S5E).

Together, this clearly shows that in the absence of any one of the components, IFNα- and IFNγ-activated responses still occur, except in IFNγ-treated STAT1 mutant cells, and that the positive feedback regulation of the ISGF3 and GAF components is preserved.

### STAT1 and STAT2 play a dual role in transcriptional regulation of GAS genes

**Fig. 4:**
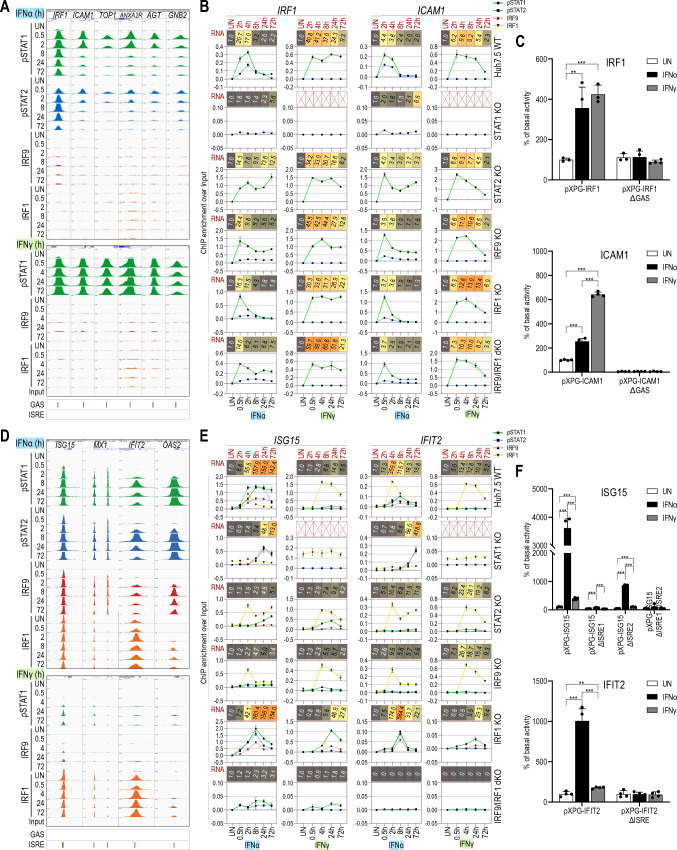
IFNα- and IFNγ-mediated induction of GAS and ISRE genes differentially depends on GAF, ISGF3 and IRF1 in a time- and phosphorylation-dependent manner. **A** Representative views of the ChIP-seq peaks detected in the promoter regions of IRF1, ICAM1, TOP1, ANXA2R, AGT and GNB2 genes, containing GAS binding sites, in untreated or treated with IFNα or IFNγ Huh7.5 cells. All peaks were mapped onto human reference genome hg38 and visualized using the IGV genome browser; scale for STATs 0–1000, IRFs 0–500; *p* phosphorylated. **B** Detailed characteristics of IRF1 and ICAM1 genes. Line charts indicated ChIP-qPCR assays performed on IRF1 and ICAM1 gene promoters in Huh7.5 wild-type or knock-out cell lines treated with IFNα or IFNγ (*n* = 2, mean ± SEM). Red boxes represent expression data from RNA-seq experiment (*n* = 2; mean fold change over untreated sample). RNA-seq experiment for Huh7.5 STAT1KO treated with IFNγ was not performed. **C** Results of luciferase-based reporter assay for IRF1 and ICAM1 promoters. Wild-type and mutated (Δ)GAS sequences of the promoter regions from IRF1 and ICAM1 were cloned into pXPG vector. Huh7.5 cells were co-transfected with pXPG (Firefly luciferase—FLUC) and pRL-SV40 (Renilla luciferase—RLUC) expression vectors and left untreated or stimulated with IFNα or IFNγ for 8 h. After harvesting, cells were lysed and the fluorescence of FLUC and RLUC levels were measured. Bars indicate the Relative Luminescence Units (RLU) of the sample calculated as a percentage of basal activity of the untreated sample transfected with pXPG vector containing wild-type sequence of the promoter. *n* = 3; mean ± SEM, *p* calculated with unpaired two-tailed Student *t* test, *p < 0.05, **p < 0.01, and ***p < 0.001; (graph shows the representative experiment). **D** Analogous graph to **A** representing ISRE-containing genes—ISG15, MX1, IFIT2 and OAS2. **E** Analogous graph to **B** for ISRE-containing genes—ISG15 and IFIT2. **F** Results of luciferase-based reporter assay for ISG15 and IFIT2 promoters. Wild-type and mutated (Δ)ISRE sequences of the promoter regions from ISG15 and IFIT2 were cloned into pXPG vector. For more details, see **C**

A striking observation after comparing IFNα and IFNγ commonly up-regulated GAS genes revealed the differential recruitment of GAF and GAF-like complexes depending on the type of IFN (Fig. 2B). Close examination of the known GAS-containing genes IRF1, ICAM1, TOP1, ANXA2R, AGT and GNB2 by ChIP-seq (Fig. 4A) and of IRF1 and ICAM1 by ChIP-PCR (Fig. 4B) confirmed a correlation between early and transient expression and recruitment of both pSTAT1 and pSTAT2 in response to IFNα, and only pSTAT1 upon IFNγ treatment (Fig. 4A, B). In line with the lack of STAT2 phosphorylation after IFNγ treatment (Fig. 1A), no recruitment of STAT2 could be detected to the IRF1 gene under these conditions as compared to IFNα treatment (Fig. S6). Site-directed mutagenesis in combination with promoter-luciferase expression analysis was used to confirm the functionality of the proximal GAS sites in the promoters of the IRF1 and ICAM1 genes (Fig. 4C). Constructs containing wild-type (WT) promoters of IRF1 and ICAM1 showed high luciferase activity in response to both IFNs. However, this effect was more prominent after treatment with IFNγ, which correlated with stronger pSTAT1 recruitment (Fig. 4A) and higher expression (Figs. 1B, 4B) after IFNγ stimulation. This effect was completely abolished by introducing mutations in the GAS sequences in both promoters.

To further characterize the dependence of IRF1 and ICAM1 on STAT1 and STAT2, we compared their expression in wt and STAT1-, STAT2-, IRF9-, IRF1- and IRF9/IRF1-mutant Huh7.5 cells (Fig. 4C). As expected, lack of STAT1 completely abrogated the expression of IRF1 and ICAM1 upon IFNγ treatment (Fig. S7). Otherwise, the expression of IRF1 and ICAM1 clearly followed the phosphorylation pattern of STAT1 and/or STAT2 in the different cell lines in response to the different types of IFN (Fig. S5). Remarkably, IFNα-induced expression was only partially lost in the STAT2-KO cells, which coincided with lower phosphorylated STAT1 levels (Fig. S5C). Moreover, their expression was not dependent on IRF9 or IRF1 (Fig. 4B). These results were further verified by quantitative ChIP-PCR, and demonstrated to correlate with binding of both pSTAT1 and pSTAT2 in response to IFNα, and only pSTAT1, but no pSTAT2, upon IFNγ treatment (Fig. 4B). Surprisingly, in the STAT1KO cells a response to IFNα could still be detected, with a clear shift in expression of these genes to later time-points (Fig. 4B). High STAT2 phosphorylation levels at later time-points (Fig. S5B), correlated with weak, but significantly increased pSTAT2 recruitment to GAS motifs in IRF1 and ICAM1 (Fig. 4B), in the absence of STAT1.

### IFNα- and IFNγ-mediated induction of ISRE genes differentially depends on ISGF3 and IRF1

In contrast to GAS genes, binding characteristics of ISRE genes clearly pointed to the regulatory role of ISGF3 together with IRF1. Close examination of the known ISRE-containing genes ISG15, MX1, IFIT12 and OAS2, by ChIP-seq (Fig. 4D) and of ISG15 and IFIT12 by ChIP-PCR (Fig. 4E) confirmed a correlation between more prolonged expression and recruitment of all ISGF3 components (pSTAT1, pSTAT2, IRF9) and IRF1 in a time-dependent manner (even after 72 h) in response to IFNα, and mainly IRF1 upon IFNγ treatment. Interestingly, IFN-dependent binding of ISGF3 components and/or IRF1 to these ISRE genes appeared later as compared to GAF and GAF-like complexes to GAS genes. In contrast to the exclusive IFN-dependent binding of ISGF3 (pSTAT1, pSTAT2 and IRF9), IRF1 exhibited binding also under basal conditions (Fig. 4D), although it varied between different genes. In line with the lack of STAT2 phosphorylation after IFNγ treatment (Fig. 1A) no recruitment of STAT2 could be detected to a number of pre-selected ISRE genes under these conditions as compared to IFNα treatment (Fig. S6).

Site-directed mutagenesis confirmed the functionality of the proximal ISRE sites in the promoters of the ISG15 and IFIT2 genes (Fig. 4F), displaying higher luciferase activity in response to IFNα, as compared to IFNγ. This correlated with the differential involvement of ISGF3 vs IRF1 (Fig. 4D) and the higher gene expression (Fig. 4E) after IFNα stimulation. This effect was completely abolished by introducing mutations in the ISRE sequences in both promoters (Fig. 4F).

Further characterization of the expression of ISG15 and IFIT2 in wt and STAT1-, STAT2-, IRF9-, IRF1- and IRF9/IRF1-mutant Huh7.5 cells (Fig. 4E), revealed a dramatic loss of IFNα-induced expression in the STAT2- and IRF9KO cells. In contrast, IFNγ-induced expression of these genes was not affected in these cell lines. As expected, lack of STAT1 completely abrogated the expression of ISG15 and IFIT2 upon IFNγ treatment (Fig. S7), whereas the IFNα response was marked by a delayed response and shift in maximum gene expression towards 72 h. Although, in IRF1KO cells IFN-induced expression of ISG15 and IFIT2 was normal, in the IRF9/IRF1KO cells, both genes responded neither to IFNα nor to IFNγ (Fig. 4E).

These results were further verified by quantitative ChIP-PCR, and demonstrated to correlate with binding of all ISGF3 components and IRF1 to ISRE motifs in ISG15 and IFIT2 in response to IFNα (Fig. 4E), and mainly IRF1, but no pSTAT1, pSTAT2 and IRF9, upon IFNγ treatment in wt cells (Fig. 4E). In IFNα-treated STAT2- and IRF9KO cells, the remaining IRF1 binding (Fig. 4E) was not able to compensate for the loss of ISGF3-dependent transcription. In contrast, IFNγ-induced binding of IRF1 in these cell lines was similar to WT cells (Fig. 4E). In STAT1KO cells, high STAT2 phosphorylation and IRF9 expression levels at later times (Fig. S5B), together with increased recruitment of pSTAT2 and IRF9 to ISRE motifs in ISG15 and IFIT2 in response to IFNα (Fig. 4E), is in agreement with the functional role of the STAT2/IRF9 complex under these conditions, as a replacement of ISGF3 activity in wt cells [44]. On the other hand, prolonged STAT1 phosphorylation and IRF9 expression levels in IRF1KO cells (Fig. S5E), together with the increased pSTAT1 and IRF9 recruitment (Fig. 4E), could point to the possible involvement of the ISGF3-like STAT1/IRF9 complex in the potent transcriptional regulation of ISRE genes in response to IFNγ [45].

### The ISRE + GAS composite site shares features of GAS and ISRE genes and acts as a molecular switch in response to IFNα and IFNγ

As mentioned above, composite genes exhibited combined features of GAS and ISRE-containing genes, with the collective recruitment of pSTAT1, pSTAT2, IRF9 and IRF1 after IFNα stimulation and pSTAT1 + IRF1 and less frequently IRF9 after IFNγ (Fig. 2B).

**Fig. 5:**
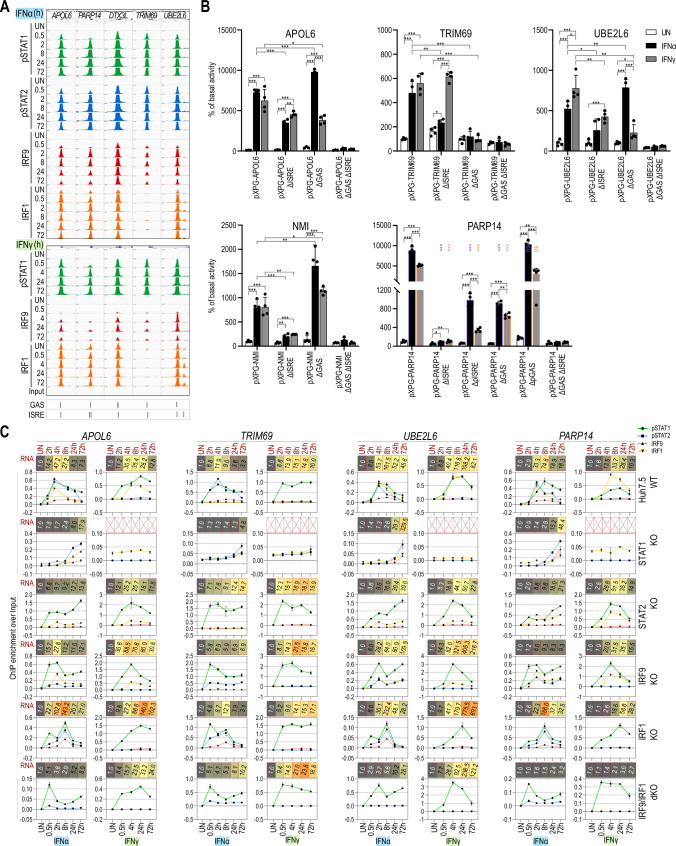
The ISRE + GAS composite site shares features of GAS and ISRE genes and acts as a molecular switch in response to IFNα and IFNγ. **A** Representative views of the ChIP-seq peaks detected in the promoter regions of APOL6, PARP14, DTX3L, TRIM69 and UBE2L6 genes, containing GAS + ISRE binding sites, in untreated or treated with IFNα or IFNγ Huh7.5 cells. All peaks were mapped onto human reference genome hg38 and visualized using the IGV genome browser; scale for STATs 0–1000, IRFs 0–500; *p* phosphorylated. **B** Results of luciferase-based reporter assay for APOL6, TRIM69, UBE2L6, NMI and PARP14 promoters. Wild-type and mutated (Δ)GAS and/or ISRE sequences of the promoter regions were cloned into pXPG vector. Huh7.5 cells were co-transfected with pXPG (Firefly luciferase—FLUC) and pRL-SV40 (Renilla luciferase—RLUC) expression vectors and left untreated or stimulated with IFNα or IFNγ for 8 h. After harvesting, cells were lysed and the fluorescence of FLUC and RLUC levels were measured. Bars indicate the Relative Luminescence Units (RLU) of the sample calculated as a percentage of basal activity of the untreated sample transfected with pXPG vector containing wild-type sequence of the promoter. *n* = 3; mean ± SEM, *p* calculated with unpaired two-tailed Student *t* test, *p < 0.05, **p < 0.01, and ***p < 0.001; To maintain clarity, we denoted statistical significance between the response of wild-type and mutated constructs in our PARP14 experiments using violet and orange stars for IFNα- and IFNγ-treated samples, respectively (graph shows the representative experiment). **C** Detailed characteristics of APOL6, PARP14, TRIM69 and UBE2L6 genes. Line charts indicated ChIP-qPCR assays performed on APOL6, PARP14, TRIM69 and UBE2L6 gene promoters in Huh7.5 wild-type or knock-out cell lines treated with IFNα or IFNγ (*n* = 2, mean ± SEM). Red boxes represent expression data from RNA-seq experiment (*n* = 2; mean fold change over untreated sample). RNA-seq experiment for Huh7.5 STAT1KO treated with IFNγ was not performed

By more detailed analysis of the IFNα and IFNγ commonly up-regulated genes, promoters of a number of pre-selected composite genes (Fig. 5A; exemplified by APOL6, PARP14, DTX3L, TRIM69, and UBE2L6) displayed a similar time-dependent binding pattern of pSTAT1, pSTAT2, IRF9 and IRF1 (except for TRIM69) in IFNα-treated cells. Likewise, binding of pSTAT1, IRF9 and IRF1 was observed, except TRIM69 (no IRF1 binding was visible), in response to IFNγ (Fig. 5A). This corresponded with the presence of an ISRE and a GAS element in close proximity in their promoters, with a random organization and varying distance (Table S4). Interestingly, IFNα-dependent binding of ISGF3 components to these composite genes appeared earlier (and stronger) as compared to ISRE genes, but was still clearly detectable after 72 h. In contrast, IFNγ-dependent binding of GAF followed a similar pattern as compared to GAS genes. IRF1 binding patterns between ISRE and composite genes were comparable. As shown for ISRE genes, IRF1 exhibited binding to composite genes already under basal conditions (Fig. 5A). In line with the lack of STAT2 phosphorylation after IFNγ treatment (Fig. 1A) no recruitment of STAT2 could be detected to a number of pre-selected composite genes under these conditions as compared to IFNα treatment (Fig. S6).

Site-directed mutagenesis in combination with promoter-luciferase expression analysis of the APOL6, UBE2L6, PARP14, NMI and TRIM69 gene promoters further highlighted the unique characteristics of composite genes in relation to the orientation and the varying distance between GAS and ISRE sites (Table S4; Fig. 5B). For example, promoter constructs for the APOL6, UBE2L6 and TRIM69genes, in which the distance between the GAS and ISRE site is similar (14–19 nt; Table S4) and orientation varies (Table S4), showed high luciferase activity in response to both IFNs, which correlated with the comparative ISGF3, GAF and IRF1 binding patterns and equal expression after IFNα and IFNγ stimulation (Fig. 5C). More interestingly, ISRE mutated APOL6, UBE2L6 and TRIM69 promoters still responded to both types of IFN, where the remaining GAS site tended to respond more to IFNγ. The opposite pattern was visible in cells expressing mutant GAS constructs for APOL6 and UBE2L6, being more sensitive to IFNα. In contrast, the GAS mutated TRIM69 promoter did not significantly respond anymore to either type of IFN. Mutations introduced simultaneously in both regulatory elements of all 3 genes resulted in the complete loss of promoter activity in response to IFNα and IFNγ (Fig. 5B). In contrast, the organization of the composite site in the PARP14 gene is characterized by a GAS site directly adjacent to the ISRE (Table S4). ISRE mutations resulted in major loss of responsiveness to IFNα and IFNγ. GAS mutations were not so drastic and the remaining ISRE site responded more to IFNα than to IFNγ. While mutations introduced simultaneously in both regulatory elements of PARP14 resulted in the complete loss of promoter activity in response to IFNα and IFNγ (Fig. 5B). The adjacent GAS-ISRE organization in PARP14 theoretically predicts the presence of an overlapping ISRE site (Table S4). Interestingly, introducing mutations only in the 3′ half of the ISRE site resulted in partial response to both types of IFN (ΔpISRE, Fig. 5B), pointing to the functionality of this overlapping ISRE. On the other hand, mutating the 5′ half of the GAS site (ΔpGAS) did not significantly affect IFNα and IFNγ responsiveness and predicted a more dominant role of the ISRE site. Finally, to assess the effect of longer spacing between the ISRE and GAS site, we further characterized the composite site organization in the NMI gene. In this case, the two sites are separated by 274 nt (Table S4). As shown for APOL6, UBE2L6 and TRIM69 promoters, the ISRE mutated NMI promoter only responded partially to both types of IFN (Fig. 5B). In contrast, mutation of the GAS site tended to increase the sensitivity to IFNα as well as IFNγ (Fig. 5B), again pointing to a more dominant role of the ISRE site. While, mutations introduced simultaneously in both GAS and ISRE elements resulted in the complete loss of IFN-dependent promoter activity.

In contrast to ISRE genes, composite genes sustained the ability to increase the expression in all mutant cell lines in response to both IFNs (Fig. 5C). Importantly, combined recruitment of pSTAT1, pSTAT2, IRF9 and IRF1 in WT cells to APOL6, UBE2L6, PARP14, and TRIM69 confirmed the importance of the ISRE site, and possibly the GAS site, after IFNα stimulation. However, collective pSTAT1 + IRF1 and weak IRF9 binding after IFNγ, clearly pointed to the combined use of the GAS + ISRE composite site under these conditions (Fig. 5C), except for TRIM69 where pSTAT1 binding (without IRF1) associated with a functional GAS site only. As expected, for all genes, no pSTAT2 binding could be detected upon IFNγ treatment.

As shown for the ISRE genes ISG15 and IFIT2, the IFNα response of APOL6, PARP14, UBE2L6 and TRIM69 in STAT1KO cells was marked by a delayed response and shift in maximum gene expression towards 72 h (Fig. 5C). Also, lower but intact IFNα-induced gene expression of APOL6, PARP14, UBE2L6 in the STAT2- and IRF9KO cells, in combination with pSTAT1 and IRF1 binding, pointed to a shift from an ISGF3/ISRE-dependent to a pSTAT1 + IRF1/composite site-dependent mechanism (Fig. 5C). For TRIM69 the absence of IRF1 binding under these conditions, pointed to a shift from an ISGF3/ISRE-dependent to a pSTAT1 + pSTAT2/GAS (in case of IFNα) or pSTAT1/GAS (in case of IFNγ) site-dependent mechanism (Fig. 5C). In the IRF1KO cells, intact expression of all genes in response to IFNα correlated with recruitment of all ISGF3 components to the ISRE site. The IFNγ response in these cells was marked by strong pSTAT1 and weak IRF9 recruitment (Fig. 5C), which could point to the sole involvement of GAF binding to the GAS or a combined involvement of GAF and STAT1/IRF9 binding to the GAS + ISRE composite site. Likewise, active IFNα and IFNγ-mediated gene expression in IRF9/IRF1KO cells, together with pre-dominant pSTAT1 recruitment, implied a shift to the independent use of the GAS site, without the neighboring ISRE site in response to both types of IFN. As expected, lack of STAT1 completely abrogated the expression of APOL6, PARP14, UBE2L6 and TRIM69 (Fig. S7) and binding upon IFNγ treatment (Fig. 5C).

These results provide further proof that both the ISRE and GAS sites present in composite genes are functional at varying distances and can be used together optimally through transcription factor co-binding or independently, and can respond to both types of IFN depending on the available components and active transcription factor complexes. Together with the presence of variable spacing between and different orientation of GAS and ISRE sites in the promoters of these composite genes (Table S4) and the sustained expression and TF binding in mutant cell lines (Fig. 5C), this predicts a common mechanism of close collaboration of GAF, ISGF3 and IRF1 complexes, without direct interaction.

### STAT1, STAT2 and IRF9 are functional composite genes that are part of a phosphorylation-dependent positive feedback loop controlling long-term IFNα and IFNγ responses

As shown in Fig. 1A, protein expression of STAT1, STAT2 and IRF9 exhibited a prolonged character and increased even after long-term treatment. This marks the positive feedback regulation of the ISGF3 and GAF components observed in response to IFNα as well as IFNγ.

**Fig. 6:**
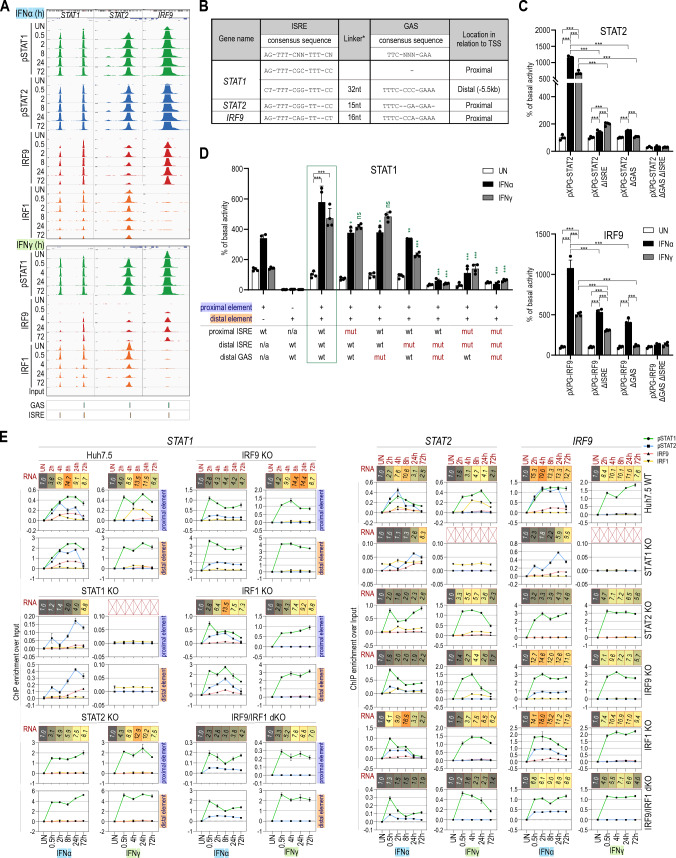
STAT1, STAT2 and IRF9 are functional composite genes that are part of a positive feedback loop controling long-term IFNα and IFNγ responses. **A** Views of the ChIP-seq peaks detected in the promoter regions of STAT1, STAT2, IRF9 genes in untreated or treated with IFNα or IFNγ Huh7.5 cells. For STAT1 in addition the distal (− 5.5 kb) regulatory element containing GAS and ISRE elements is visualized. All peaks were mapped onto human reference genome hg38 and visualized using the IGV genome browser; scale for STATs 0–1000, IRFs 0–500; *p* phosphorylated. **B** Regulatory elements characterized in the STAT1, STAT2 and IRF9 genes. Sequences characterized in the promoters of STAT1, STAT2 and IRF9 together with the distal regulatory element of STAT1. *Distance between two elements, *nt* nucleotide, References see in the text. **C** Results of luciferase-based reporter assay for STAT2 and IRF9 promoters. Wild-type and mutated (Δ)GAS and/or ISRE sequences of the promoter regions were cloned into pXPG vector. Huh7.5 cells were co-transfected with pXPG (Firefly luciferase—FLUC) and pRL-SV40 (Renilla luciferase—RLUC) expression vectors and left untreated or stimulated with IFNα or IFNγ for 8 h. After harvesting, cells were lysed and the fluorescence of FLUC and RLUC levels were measured. Bars indicate the Relative Luminescence Units (RLU) of the sample calculated as a percentage of basal activity of the untreated sample transfected with pXPG vector containing wild-type sequence of the promoter. *n* = 3; mean ± SEM, *p* calculated with unpaired two-tailed Student *t* test, *p < 0.05, **p < 0.01, and ***p < 0.001 (graph shows the representative experiment). **D** Results of luciferase-based reporter assay for STAT1 promoter and/or distal regulatory element. Wild-type and mutated (Δ)GAS and/or ISRE sequences of the promoter regions with/without distal (− 5.5 kb) regulatory element were cloned into pXPG vector; *n* = 3; mean ± SEM, green box indicated the reference construct containing wild-type sequences of proximal promoter and distal regulatory element; statistics was calculated between analogous samples between reference and experimental constructs; *p* calculated with Student *t* test, *p < 0.05, **p < 0.01, and ***p < 0.001 (graph shows the representative experiment). For more detailed description of experimental procedure, see **B**. **E** Detailed characteristics of STAT1, STAT2 and IRF9 genes. Line charts indicated ChIP-qPCR assays performed on gene proximal promoter or distal (− 5.5 kb) regulatory element in Huh7.5 wild-type or knock-out cell lines treated with IFNα or IFNγ (*n* = 2, mean ± SEM). Red boxes represent expression data from RNA-seq experiment (*n* = 2; mean fold change over untreated sample). RNA-seq experiment for Huh7.5 STAT1KO treated with IFNγ was not performed

Close examination of the STAT1, STAT2 and IRF9 genes by ChIP-seq (Fig. 6A), revealed a similar binding pattern as seen for composite genes (Fig. 5A). Indeed, binding of pSTAT1, pSTAT2, IRF9 and IRF1, was observed to the promoter of *STAT1* and *STAT2* in a time-dependent manner (even after 72 h) in IFNα-treated cells and pSTAT1 and IRF1 and weak IRF9 to these genes in response to IFNγ (Fig. 6A). The same was true for *IRF9*, however under these conditions IRF1 binding could not be detected (Fig. 6A). These binding characteristics for the STAT1, STAT2 and IRF9 genes corresponded with the presence of an ISRE and a GAS element in close proximity in the promoters of *STAT2* and *IRF9*, and an ISRE in the proximal *STAT1* promoter, combined with a distal ISRE and GAS composite site (Fig. 6B). In line with the lack of STAT2 phosphorylation after IFNγ treatment (Fig. 1A), no recruitment of STAT2 could be detected to the STAT1, STAT2 and IRF9 genes under these conditions as compared to IFNα treatment (Fig. S6).

Next, site-directed mutagenesis confirmed the functionality of the proximal ISRE and GAS sites in the promoters of the *STAT2* and *IRF9* genes (Fig. 6C). To study the functionality of the proximal ISRE site and the distal composite site in the STAT1 gene (Fig. 6B), we first compared constructs containing the proximal and distal fragments separately or in combination (Fig. 6D). Clearly, both the proximal ISRE site and the distal composite site were required for an optimal response to both IFNα and IFNγ. This was confirmed by subsequent mutation analysis of individual or combined ISRE and GAS sites. Mutations introduced simultaneously in all three regulatory elements resulted in the complete loss of *STAT1* promoter activity in response to IFNα and IFNγ (Fig. 6D).

Remarkably, IFN-induced expression of *STAT1*, *STAT2* and *IRF9* was present in all mutant cell lines (except in IFNγ-treated STAT1KO cells: Figs. S5 and S7) (Fig. 6E), which resembles the characteristics of composite-containing genes. More important, it marked the restoration of the positive feedback regulation loop of ISGF3 and GAF components under these conditions. Subsequent quantitative ChIP-PCR further confirmed these observations (Fig. 6E), with combined recruitment of pSTAT1, pSTAT2 and IRF9 with (*STAT1*prox, *STAT1*dist and *STAT2*) or without (*IRF9*) IRF1 in WT cells after IFNα stimulation. Likewise, collective pSTAT1 + IRF1 and weak IRF9 binding could be observed after IFNγ (Fig. 6E), except for *IRF9* where only pSTAT1 binding (without IRF1) was detected. As expected, for all three genes no pSTAT2 binding could be detected upon IFNγ treatment.

Also, the IFNα response of *STAT1*, *STAT2* and *IRF9* in STAT1KO cells was marked by a delayed response and shift in maximum gene expression towards 72 h (Fig. 6E). Moreover, lower but intact IFNα-induced gene expression of *STAT1* and *STAT2* in the STAT2- and IRF9KO cells, in combination with pSTAT1 (and sometimes pSTAT2) and IRF1 binding, pointed to a shift from an ISGF3/ISRE dependent to a pSTAT1 + IRF1/composite site-dependent mechanism. For *IRF9,* the absence of IRF1 binding under these conditions pointed to a shift from an ISGF3/ISRE-dependent to a pSTAT1 + pSTAT2/GAS (in case of IFNα) or pSTAT1/GAS (in case of IFNγ) site-dependent mechanism. In the IRF1KO cells, intact expression of all three genes in response to IFNα correlated with recruitment of all ISGF3 components to the ISRE site. The IFNγ response of *STAT1*, *STAT2* and *IRF9* in these cells was marked by strong pSTAT1 and weak IRF9 recruitment (Fig. 6E), which could point to the sole involvement of GAF binding to the GAS or a combined involvement of GAF and STAT1/IRF9 binding to the GAS + ISRE composite site. Finally, active IFNα and IFNγ-mediated expression of these genes in IRF9/IRF1KO cells, together with pre-dominant pSTAT1 recruitment, implied a shift to the independent use of the GAS site, without the neighboring ISRE site in response to both types of IFN (Fig. 6E).

## Discussion

Historically, IFNα signaling was perceived as a pre-dominant ISRE-dependent ISGF3-mediated response [46] and IRF1 [47, 48]. In contrast, IFNγ responses were mainly characterized as GAS–GAF driven [12, 49]. Over the years, however, evidence emerged that IFNα and IFNγ induce an overlapping set of TFs, i.e., complexes containing phosphorylated STAT1 and STAT2, IRF9 and IRF1 [5, 6, 50, 51]. In our recently published Hypothesis and Theory article, closer inspection of the publicly accessible dataset [5, 52] featuring STAT1, STAT2, and IRF1 ChIP-seq experiments performed on chromatin extracted from short-term IFNα- or IFNγ-treated K562 cells (http://www.encodeproject.org), could recognize the binding to typical ISRE or GAS-containing genes, but also to ISRE and GAS composite sites. Together with the results from our integrative analysis this offers clear evidence for the existence of a GAS + ISRE composite gene group, which together with GAS and ISRE genes are commonly involved in IFNα- and IFNγ-mediated transcriptional responses through recruitment of STAT1- and STAT2-containing ISGF3 and GAF-like complexes and IRF1 in a time- and phosphorylation-dependent manner.

Accordingly, early expression of GAS genes was supported by early and transient binding of pSTAT1 alone (GAF) or pSTAT1 together with pSTAT2 (GAF-like) in response to IFNα. This coincided with individual or combined roles of GAF and GAF-like complexes and corresponded with the presence of a single functional GAS element and no ISRE in their proximal promoter. Upon IFNγ treatment only pSTAT1 was recruited, which was stronger and more prolonged and pointed to the sole role of GAF. This also correlated with the prolonged expression of GAS genes in response to IFNγ. In contrast, later expression of ISRE genes in response to both types of IFN correlated with collective recruitment of all ISGF3 components (pSTAT1, pSTAT2, IRF9) with or without IRF1, or only IRF1 in response to IFNα. IFNγ predominantly directed IRF1 binding to these genes, but less frequently and with lower affinity also IRF9 and pSTAT1. This corresponded with the presence of a functional ISRE element and no GAS in their proximal promoter. In addition, it further highlighted the exclusive IFN-dependent binding of ISGF3 (pSTAT1, pSTAT2 and IRF9) as opposed to IRF1 which exhibited binding to these ISRE genes also under basal conditions. Obviously, IFN-dependent binding of ISGF3 components and/or IRF1 to these ISRE genes appeared later as compared to GAF and GAF-like complexes to GAS genes. Moreover this binding to ISRE genes was still clearly detectable after 72 h and correlated with long-term ISG expression. This pointed to the later recruitment of ISGF3 and IRF1 to ISRE sites and correlated with the time-dependent transcriptional differences of GAS (early) and ISRE (later) genes. Stronger binding of ISGF3 components to ISRE sites as compared to GAS, reflected the potency of ISGF3 and the higher induction of ISRE genes as compared to GAS genes.

Finally, the newly identified group of composite genes displayed a more heterogenous expression profile in response to both types of IFN, resembling that of GAS (Early) and ISRE (Later) genes. The combined recruitment of pSTAT1, pSTAT2, IRF9 and IRF1 after IFNα stimulation and pSTAT1 + IRF1 and less frequently IRF9 after IFNγ correlated with the close proximity of GAS and ISRE sites, and revealed combined binding features of GAS and ISRE-containing genes. IFNα-dependent binding of ISGF3 components to these composite genes appeared earlier (and stronger) as compared to ISRE genes, and also correlated with the more dominant role of the ISRE site as compared to the GAS site under these conditions. Our data are, therefore, suggestive of a minor role of GAF or GAF-like complexes in early activation followed by a major role of ISGF3 and IRF1 in maintaining long-term IFNα-activated expression of composite genes. On the other hand, IFNγ-activated long-term transcriptional responses of composite genes clearly depended on both GAS and ISRE sites, directed by time-dependent recruitment of GAF and IRF1. Together, this could explain the more heterogeneous nature of IFN-dependent composite gene expression, being later (and more prolonged) than GAS genes upon IFNα treatment and earlier than ISRE genes in IFNγ-treated cells.

More important, our results provide further proof that ISRE and GAS sites in composite genes are functionally important, either alone or in a collaborative fashion, in response to both types of IFN through co-binding of GAF, GAF-like, ISGF3 and IRF1 complexes. Technical limitations of our ChIPseq experiments, however, can’t rule out the existence of gene fractions that are occupied by only one TF and not the other. The mechanism of STAT and IRF co-binding in transcriptional regulation of ISRE and GAS-containing ISGs is a known phenomenon. For example, both STAT1 and IRF1 were shown to control the IFNγ-induced expression of CIITA, GBP1, and gp19 [10, 13, 53]. Likewise, STAT1–IRF1 co-binding generally occurred in an independent study of 128 transcription factors in IFNγ-treated K562 cells [54]. Hassan et al. further highlighted the importance of STAT1 and IRF1 cooperation by detailed studies of ISG-rich chromosomal segments in HeLa cells [55]. Likewise, co-binding of STAT1-containing transcription factor complexes and NFkB, activated by IFN-I or IFN-II together with LPS, provides a platform for robust transcriptional activation of pro-inflammatory genes [20]. In this respect, STAT1 and p65 co-binding correlated with the close proximity of GAS and NFkB (41–234 bp) or ISRE and NFkB (38–264 bp) binding sites [20]. This STAT1-p65 co-binding correlated with histone acetylation, PolII recruitment, and resulted in maximum target gene transcription in a STAT1-p65 co-bound dependent manner. A similar organization of closely located ISRE and NFkB sites, within ~ 50 bp proximity, was reported for IRF3 and NFkB co-occupancy to control Sendai virus-induced gene activation [56].

Our integrative analysis also resulted in the identification of a selective group of IFNα- and IFNγ-specific genes, which in general displayed lower expression and weaker binding characteristics as compared to the common genes. Interestingly, the main function of these IFNα- and IFNγ-specific genes were connected to the defence response and immune system process, and in case of IFNγ-specific genes also to RNA biosynthetic process. Based on the presence of the binding site and the binding pattern of pSTAT1, pSTAT2, IRF9 and/or IRF1, it is tempting to speculate that IFNα-specific gene regulation involves both ISRE- and GAS-dependent mechanisms, whereas IFNγ-specific transcription tends to be more GAS-GAF-driven. In this respect, we cannot rule out the involvement of other STATs and/or IRFs. On the other hand, STAT- and IRF-independent mechanisms or IFNα- and IFNγ-specific changes in chromatin remodeling/histone modifications [6] can also be involved. Further experimental proof for this needs to be provided, but is outside the scope of this study.

Close examination of gene expression in combination with chromatin binding characteristics, in wt and STAT1-, STAT2-, IRF9-, IRF1- and IRF9/IRF1-mutant Huh7.5 cells, further supported a number of additional conclusions connected to the transcriptional regulation of GAS, ISRE and composite genes. First, in addition to the accepted role of GAF (pSTAT1 homodimers), our results are in line with a novel role of a pSTAT1–pSTAT2 GAF-like complex in the transcriptional response of GAS genes to IFNα [5]. Comparative analysis of RNAseq and ChIPseq data from IFNβ- and IFNγ-treated mouse wt and IRF9-KO macrophages [52, 57] confirmed these observations (not shown). In other studies [7, 58], a heterodimer of STAT1 and STAT2 was shown to bind to the GAS site in the IRF1 gene [59] and regulate its expression in response to IFN-I. More important, under these conditions the STAT1–STAT2 heterodimer was identified as a stronger binding complex and proposed to be a more potent inducer than the STAT1 homodimer. Our data together with Ho et al. [60] would favor a mechanism in which formation of IFNα-activated GAF and GAF-like complexes from pSTAT1 and pSTAT2, compete for binding to GAS sites and for regulating transcription of GAS genes. At the same time they compete in the formation of ISGF3 from GAF-like complexes together with IRF9 and the transcription of ISRE genes. This could coincide with the weaker and more transient nature of GAS gene expression in response to IFNα as compared to IFNγ, in which the sole recruitment of GAF was stronger and more prolonged. Along the same lines, residual expression of GAS genes combined with recruitment of pSTAT2 in STAT1KO cells in response IFNα, could be connected to a potential regulatory role of STAT2 homodimers [61, 62]. However, additional experimental proof for this needs to be provided.

Second, in IFNα-treated STAT1KO cells, delayed transcription of ISRE and composite genes correlated with a functional role of the STAT2/IRF9 complex under these conditions as a replacement of ISGF3 activity in wt cells [63]. Thus, STAT2/IRF9 was shown to exhibit a biological function in the reconstitution of the antiviral response in cells lacking STAT1. In line with this, Yamauchi et al. [17] recently showed in Huh-7.5 cells that IFN-I responses were only partially attenuated by knock-out of STAT1 but completely by knock-out of STAT2. Moreover, they observed that IFN-I inhibited hepatitis C virus (HCV) replication in a STAT2-dependent but STAT1-independent manner [17]. Likewise, prolonged STAT1 phosphorylation and IRF9 expression levels in IFNγ-treated IRF1KO cells, together with the increased pSTAT1 and IRF9 recruitment, could point to the possible involvement of the ISGF3-like STAT1/IRF9 complex [45] or ISGF3II [16] in the transcriptional regulation of ISRE and composite genes. This shows that non-canonical ISGF3-like complexes are potentially able to restore IFN-induced transcription of ISRE and composite genes and to compensate for classical ISGF3 activity.

Third, in contrast to GAS and ISRE genes, composite genes were able to sustain IFN responsiveness in all mutant cell lines as compared to Wt cells. Collectively, it shows that the ISRE + GAS composite site shares features of GAS and ISRE genes and is able to act as a molecular switch, which depends on the combinatorial action of canonical and non-canonical ISGF3 and GAF complexes and IRF1. The involvement of other STATs and/or IRFs as compensatory mechanisms cannot be ruled out, however. Nevertheless, this highlights the flexibility of the composite site in the transcriptional response to both IFNα and IFNγ as compared to individual GAS and ISRE genes, which correlates with their conserved role in the immune response [64]. More important, it would allow for the generation of a backup response against viruses that impede STAT1, STAT2 and IRF9 activity as a major mechanism to evade antiviral responses [65–68].

Fourth, comparing the proximal promoters of the common composite genes identified in our study, revealed a random organization (ISRE-GAS or GAS-ISRE), with a distance varying between 0 and 415 bp (Table S4) [5]. In agreement with other studies, this close binding sites distribution may be a pre-requisite for effective collaboration of GAF, GAF-like, ISGF3 and IRF1 complexes in IFN-activated responses. Together with the presence of variable spacing between GAS and ISRE sites of these composite genes and the sustained expression and TF binding in mutant cell lines, predicts a common mechanism of close collaboration of GAF, ISGF3 and IRF1 complexes, without direct interaction. Moreover, it is tempting to speculate that STAT-dependent chromatin remodeling [69] and epigenetic changes are involved in time-dependent recruitment of GAF and GAF-like complexes to GAS (earlier) and ISGF3 and IRF1 to ISRE sites (later), to regulate maximum expression of composite genes in response to IFNs.

Detailed assessment of the STAT1, STAT2 and IRF9 genes by ChIP-seq, recognized a similar binding pattern as seen for composite genes and revealed novel insights into the autoregulatory mechanisms of IFN-dependent transcription of these genes. The binding characteristics for the STAT1, STAT2 and IRF9 genes corresponded with the presence of a functional ISRE and a GAS element in close proximity in the promoters of STAT2 and IRF9, and an ISRE in the STAT1 gene. Recently, a novel distal regulatory element was described positioned 5.5-kb upstream of the mouse STAT1 gene [70] with a similar ISRE and GAS composite structure as we observed in the human gene. Interestingly, this distal region displayed a similar binding pattern as seen for the proximal STAT1 promoter region, and provides proof for the presence of a composite site, in addition to the proximal ISRE site, for the human STAT1 gene comparable to its mouse homolog. The ChIP-seq data investigation of K562 cells (see above) are in agreement with this idea [5]. In analogy to the mouse gene [70], our data suggested the presence of an active chromatin looping mechanism connecting both regions and mediating maximum IFN-induced transcription of the STAT1 gene. Remarkably, IFN-induced expression of STAT1, STAT2 and IRF9 was present in all mutant cell lines (except in IFNγ-treated STAT1KO cells). This further validates the functionality of the ISRE + GAS composite site in the regulatory regions of these genes, and the ability to act as a molecular/regulatory switch in combination with canonical and non-canonical ISGF3 and GAF complexes and IRF1. It also shows that in the absence of any one of the components, the positive feedback regulation of the ISGF3 and GAF components is preserved. In addition to the fact that this positive feedback system is absent in STAT1 KO U3A cells rescued with tyrosine 701 mutated STAT1 (Y to F) [15] or STAT1 KO mice overexpressing this STAT1 Y to F mutant [71], our data agree with a model in which the IFN-dependent positive feedback regulation of STAT1, STAT2 and IRF9 depends on phosphorylated ISGF3 and GAF components.

As potential IRF1 target genes, the feedback regulation of ISGF3 and GAF components also depends on IRF1 [50]. Therefore, on the one hand positive feedback regulation of the STAT1, STAT2, IRF9 and IRF1 genes increases their protein expression, further enhancing complex formation, in an IFN- and phosphorylation-dependent manner. On the other hand, these complexes participate in the time-dependent expression of GAS, ISRE and composite ISGs in response to both types of IFN. At the same time, our results also point to the functional overlap between IFNα and IFNγ with a prominent role of the innate response at the early phase (reflected by the expression of GAS genes) followed by the contribution of the adaptive response at later phases (reflected by the expression of composite and ISRE genes). This adds a novel level to the timely steps that take place during long-term cellular responsiveness to IFN-I and IFN-II and offer an explanation for the existing molecular and functional overlap between IFN-I- and IFN-II-activated ISG expression.

IFNγ-mediated tyrosine phosphorylation of STAT2 was reported in a study using IFNγ-treated mouse primary embryonic fibroblasts that resulted in the formation of ISGF3 [50, 72]. Moreover, mice lacking IRF9 were impaired not only in their IFN-I response, but also in their IFNγ -induced ISRE-dependent gene expression [50]. Similarly in MEFs, STAT2 phosphorylation appeared to be essential for the antiviral potency of IFNγ [73]. Recently, Platanitis et al. provided evidence for a more genome-wide role of ISGF3 in responses of mouse Macrophages and fibroblasts to both IFN types [57]. Likewise, simultaneous recruitment of pSTAT1, pSTAT2 and IRF9 to classical ISRE-containing genes after IFN-I and IFNγ treatment was observed by our group in mouse VSMC and Macrophages [20]. Together, this points to the existence of an ISGF3-dependent mechanism by which IFNγ can elicit activities in different mouse cell types. In contrast, currently, there is no evidence of this phenomenon in human cells, which is in agreement with the lack of STAT2 phosphorylation after IFNγ treatment of Huh7.5 cells in our study and the absence of STAT2 recruitment to GAS, ISRE and composite genes. Also, our data do not agree with the observation of Morrow et al., who provided evidence of an ISGF3 complex containing unphosphorylated STAT2 (ISGF3^II^) in IFNγ-stimulated human A549 cells with a role in antiviral activity [16].

Literature describes the involvement of unphosphorylated ISGF3 (U-ISGF3) or U-GAF in long-term IFN responses, where increased levels of ISGF3 components are no longer phosphorylated [74, 75]. In this respect, a switch is proposed from ISGF3 to U-ISGF3, which controls prolonged expression of STAT1, STAT2, and IRF9 and a diversity of other ISRE-containing ISGs. Likewise, a GAF to U-STAT1 switch can be imagined [5, 63]. According to our data, chromatin recruitment of pSTAT1 and pSTAT2 in response to IFNα was still detectable after 72 h, which would match with a time-dependent role of classical ISGF3 (pSTAT1 + pSTAT2 + IRF9) in early and prolonged ISG expression. The same would account for GAF (a homodimer of pSTAT1) in timely IFNγ responses. This is in line with detectable phosphorylation of STATs in other studies [16, 18]. Consequently, our data do not agree with this switch model. However, increased protein expression and potential binding of STAT1, STAT2 and IRF9 at later time-points cannot rule out a supplemental role of U-ISGF3 (U-STAT1 + U-STAT2 + IRF9) and U-STAT1 in prolonged ISG expression in Huh7.5 cells.

An additional role of U-ISGF3 [76] and STAT2/IRF9 [57] has also been proposed in basal ISRE-dependent transcription. Accordingly, Platanitis proposed the existence of a molecular switch from STAT2/IRF9 to ISGF3 that underlies IFN-induced transcription in mouse cells, but not in human [57]. With the absence of IRF9 binding in untreated cells our data do not favor a role of U-ISGF3 under basal conditions in Huh-7.5 cells. However, given that antibodies were used, which only recognize tyrosine phosphorylated STAT1 and STAT2, and that IRF9 amounts in untreated cells were below the level of WB detection, this model can’t be ruled out. Nevertheless, basal binding of IRF1, combined with IFN-induced recruitment to ISRE and composite genes, predicts a dual role of IRF1 in ISRE-dependent transcription. Indeed, previous studies agree with a role of IRF1 in basal and IFN-induced transcription of ISRE genes [50, 55, 77, 78]. Hence, based on our observations of IRF1 basal binding followed by IFNα-induced co-binding with ISGF3, it is tempting to speculate that a similar molecular switch exists from IRF1 to ISGF3 that underlies IFN-induced transcription of ISRE and composite genes in human cells. More important, IRF1 recruitment under basal conditions could potentially flag the genome, to facilitate rapid and specific IFN-I- and IFN-II-dependent binding of ISGF3, GAF, and/or IRF1 complexes and mediate a robust and effective antiviral response. However, additional experimental proof for this needs to be provided.

### Supplementary Information

Below is the link to the electronic supplementary material.

Figure S1. Global distribution of pSTAT1, pSTAT2, IRF9 and IRF1 binding sites in response to IFNα and IFNγ stimulation. (A,B) Global distribution of peaks annotated for pSTAT1, pSTAT2, IRF9 and IRF1 after IFNα (A) and pSTAT1, IRF9 and IRF1 after IFNγ (B) stimulation. The number assigned to each bar on the right side presents the total number of identified binding regions. Binding sites annotation to the categories of genomic localizations were performed on the lists of all non-redundant peaks combined from all time-points (0-72h) for each antibody. (C, D) Genomic localization of occupied binding sites with identified motifs GAS and/or ISRE in response to IFNα (C) or IFNγ (D) stimulation. The number on the right side represents the total number of identified binding regions with the specific motif. The number in bold indicate the most abundant motif identified in the regions occupied by the given signaling protein. (PDF 1366 KB)

Figure S2. GAS matrices used for binding sites annotation in the peak regions from ChIP-seq experiments. 10 matrices representing GAS binding site, 6 retrieved from HOMER database and 4 calculated deNovo using HOMER on pSTAT1 ChIP-seq data, were used in the first binding site annotation in the peak regions from the ChIP-seq experiment with pSTAT1 antibody. The UpSetR plot presents the number of motifs recognized with the intersections between matrices. Matrices selected for the final TF binding sites annotation based on the number of motifs recognized commonly with other matrices, as well uniquely by each matrix separately. (PDF 1612 KB)

Figure S3. ISRE matrices used for binding sites annotation in the peak regions from ChIP-seq experiments. 8 matrices representing ISRE binding site, 7 retrieved from HOMER database and 1 calculated deNovo using HOMER on pSTAT1 ChIP-seq data, were used in the first binding site annotation in the peak regions from the ChIP-seq experiment with pSTAT1 antibody. The UpSetR plot presents the number of motifs recognized with the intersections between matrices. Matrices selected for the final TF binding sites annotation based on the number of motifs recognized commonly with other matrices, as well uniquely by each matrix separately. (PDF 1551 KB)

Figure S4. Data integration and filtering pipeline. An integrative analysis of our RNAseq-ChIPseq data performed, using the BETA tool (see Experimental Model and Subject Details). By concentrating on the promoter/5’UTR regions with annotated GAS and/or ISRE motifs, our multi-omics data integration approach identified IFNa and IFNg commonly induced genes that bound any of the 4 components individually or in a random combination in their promoter region. (PDF 1359 KB)

Figure S5. Characterization of IFNα- and IFNγ-stimulated responses in WT, STAT1-, STAT2-, IRF9-, IRF1KO and IRF9/IRF1 dKO Huh7.5 cell lines. (A) WT, (B) STAT1-, (C) STAT2-, (D) IRF9-, (E) IRF1KO and (F) IRF9/IRF1 dKO Huh7.5 cells were treated with IFNα (1000 U/ml) or IFNγ (10 ng/ml) for the indicated time. The expression levels of STAT1, STAT2, IRF9 and IRF1 were evaluated by immunoblotting; p-phosphorylated, t- total. (PDF 2335 KB)

Figure S6. pSTAT1 and STAT2 recruitment to GAS, ISRE and composite genes in IFNα and IFNγ treated Hu 7.5 cells. Views of the ChIP-seq peaks detected in the promoter regions of pre-selected GAS, ISRE and composite genes in untreated or IFNα and IFNγ treated Huh7.5 cells. All peaks were mapped onto human reference genome hg38 and visualized using the IGV genome browser; scale for pSTAT1 and STAT2 0-1000. (PDF 128 KB)

Figure S7. Expression of GAS, ISRE and composite-containing representative genes in STAT1KO Huh7.5 cells after IFNγ treatment. GAS (IRF1, ICAM1), ISRE (ISG15, IFIT2) and composite (APOL6, DTX3L, STAT1, STAT2, IRF9) gene expression was analyzed using qPCR. Mean +/- SEM, n=2. (PDF 32 KB)

Table S1. Primer sequences for RT-PCR and cloning. (DOCX 19 KB)

Table S2. List of IFNα- and IFNγ-specific integrated genes. (DOCX 24 KB)

Table S3. List of IFNα- and IFNγ-common integrated GAS, ISRE and composite genes. (DOCX 19 KB)

Table S4. List of IFNα- and IFNγ-common integrated composite genes with spacing between and orientation of GAS and ISRE sites. (DOCX 19 KB)

## Data Availability

RNA sequencing and ChIP sequencing data are available at NCBI GEO DataSets under accession number: SuperSeries GSE222668.
