# Supplementary material for: Time-dependent recruitment of GAF, ISGF3 and IRF1 complexes shapes IFNα and IFNγ-activated transcriptional responses and explains mechanistic and functional overlap
Source: Cell Mol Life Sci. 2023 Jun 22;80(7):187. doi: 10.1007/s00018-023-04830-8 (PMC10287828; doi:10.1007/s00018-023-04830-8)
Supplement: Supplementary file 1 — Figure S1. Global distribution of pSTAT1, pSTAT2, IRF9 and IRF1 binding sites in response to IFNα and IFNγ stimulation. (A,B) Global distribution of peaks annotated for pSTAT1, pSTAT2, IRF9 and IRF1 after IFNα (A) and pSTAT1, IRF9 and IRF1 after IFNγ (B) stimulation. The number assigned to each bar on the right side presents the total number of identified binding regions. Binding sites annotation to the categories of genomic localizations were performed on the lists of all non-redundant peaks combined from all time-points (0-72h) for each antibody. (C, D) Genomic localization of occupied binding sites with identified motifs GAS and/or ISRE in response to IFNα (C) or IFNγ (D) stimulation. The number on the right side represents the total number of identified binding regions with the specific motif. The number in bold indicate the most abundant motif identified in the regions occupied by the given signaling protein. (PDF 1366 KB) [file 18_2023_4830_MOESM1_ESM.pdf]

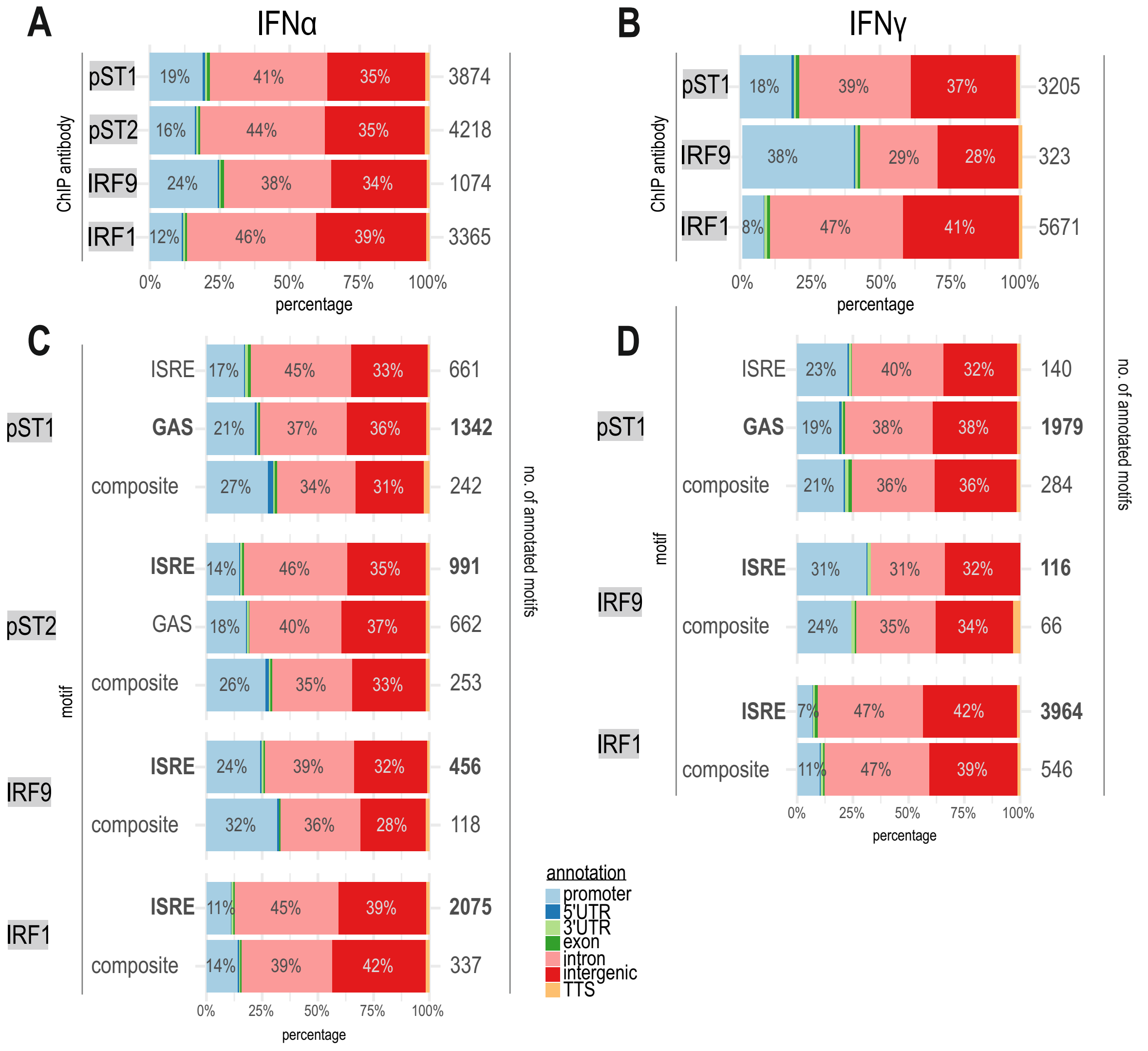
A
IFNα
B
IFNγ
C
D
ChIP antibody
pST1
pST2
IRF9
IRF1
19%
41%
35%
3874
16%
44%
35%
4218
24%
38%
34%
1074
12%
46%
39%
3365
18%
39%
37%
3205
38%
29%
28%
323
8%
47%
41%
5671
0%
25%
50%
75%
100%
percentage
motif
ISRE
GAS
composite
no. of annotated motifs
17%
45%
33%
661
21%
37%
36%
1342
27%
34%
31%
242
14%
46%
35%
991
18%
40%
37%
662
26%
35%
33%
253
24%
39%
32%
456
32%
36%
28%
118
11%
45%
39%
2075
14%
39%
42%
337
23%
40%
32%
140
19%
38%
38%
1979
21%
36%
36%
284
31%
31%
32%
116
24%
35%
34%
66
7%
47%
42%
3964
11%
47%
39%
546
annotation
promoter
5'UTR
3'UTR
exon
intron
intergenic
TTS
