# Supplementary figures and images for: Time-dependent recruitment of GAF, ISGF3 and IRF1 complexes shapes IFNα and IFNγ-activated transcriptional responses and explains mechanistic and functional overlap
Source: Cell Mol Life Sci. 2023 Jun 22;80(7):187. doi: 10.1007/s00018-023-04830-8 (PMC10287828; doi:10.1007/s00018-023-04830-8)

A

## GAS - all matrices

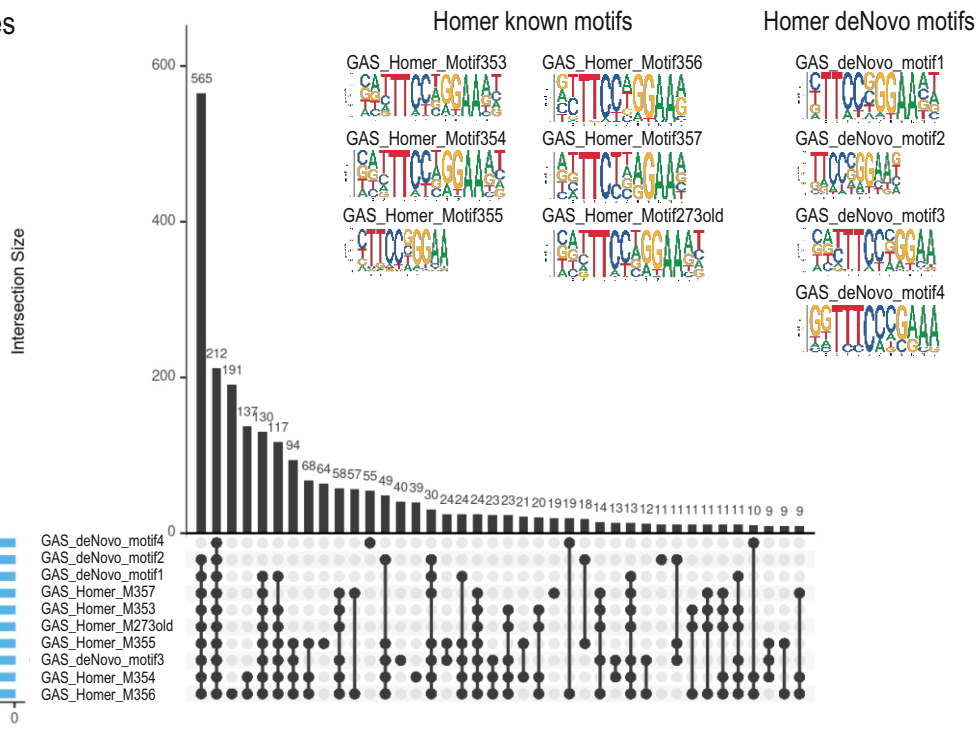

B

## GAS - selected matrices

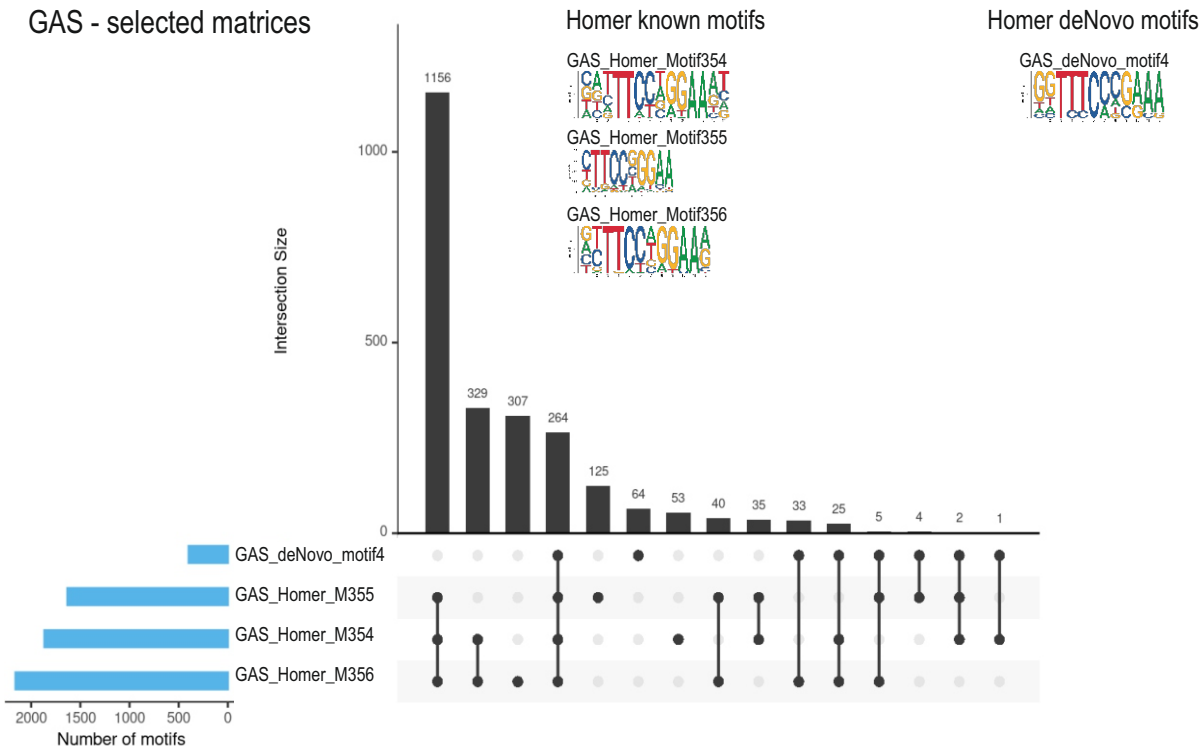

Supplement: Supplementary file 2 — Figure S2. GAS matrices used for binding sites annotation in the peak regions from ChIP-seq experiments. 10 matrices representing GAS binding site, 6 retrieved from HOMER database and 4 calculated deNovo using HOMER on pSTAT1 ChIP-seq data, were used in the first binding site annotation in the peak regions from the ChIP-seq experiment with pSTAT1 antibody. The UpSetR plot presents the number of motifs recognized with the intersections between matrices. Matrices selected for the final TF binding sites annotation based on the number of motifs recognized commonly with other matrices, as well uniquely by each matrix separately. (PDF 1612 KB) [file 18_2023_4830_MOESM2_ESM.pdf]

A

## ISRE - all matrices

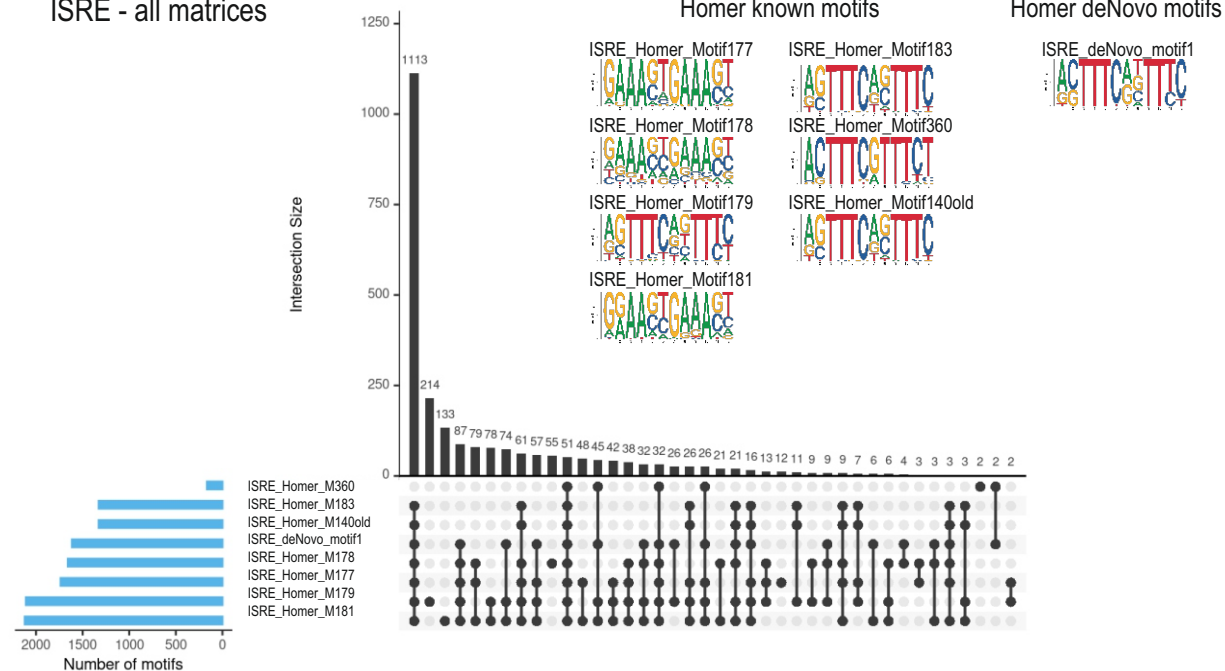

B

## ISRE - selected matrices

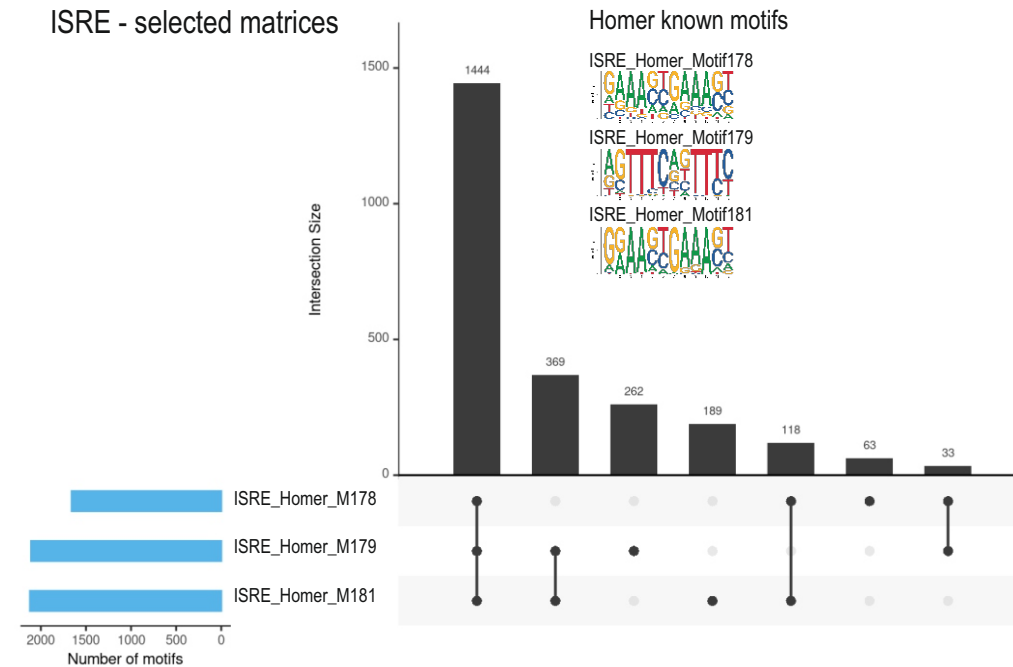

Supplement: Supplementary file 3 — Figure S3. ISRE matrices used for binding sites annotation in the peak regions from ChIP-seq experiments. 8 matrices representing ISRE binding site, 7 retrieved from HOMER database and 1 calculated deNovo using HOMER on pSTAT1 ChIP-seq data, were used in the first binding site annotation in the peak regions from the ChIP-seq experiment with pSTAT1 antibody. The UpSetR plot presents the number of motifs recognized with the intersections between matrices. Matrices selected for the final TF binding sites annotation based on the number of motifs recognized commonly with other matrices, as well uniquely by each matrix separately. (PDF 1551 KB) [file 18_2023_4830_MOESM3_ESM.pdf]

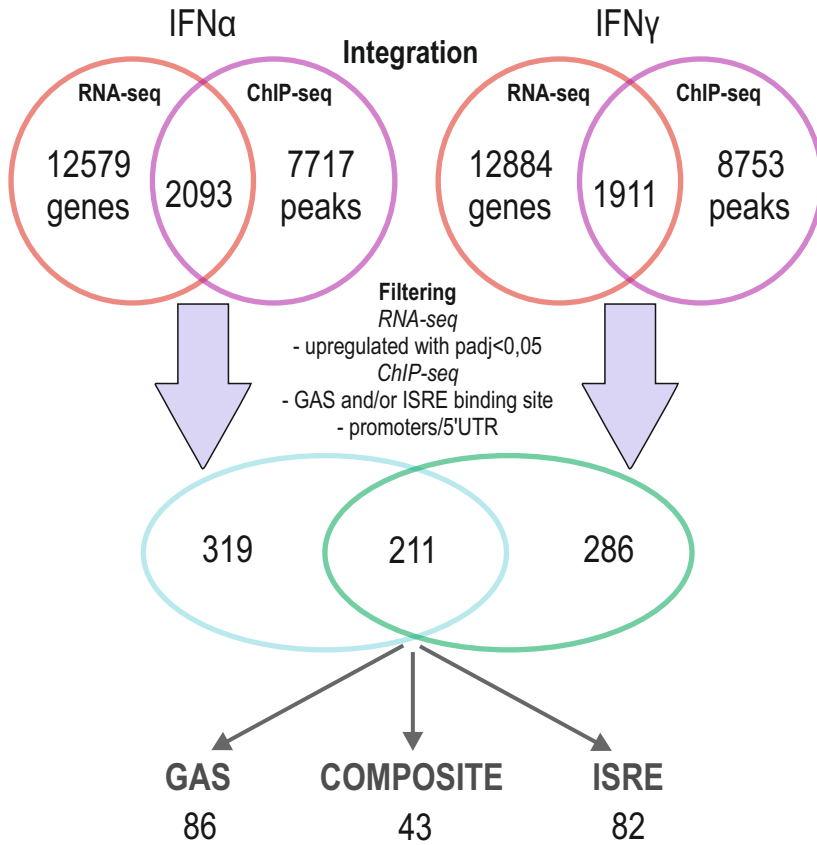

Supplement: Supplementary file 4 — Figure S4. Data integration and filtering pipeline. An integrative analysis of our RNAseq-ChIPseq data performed, using the BETA tool (see Experimental Model and Subject Details). By concentrating on the promoter/5’UTR regions with annotated GAS and/or ISRE motifs, our multi-omics data integration approach identified IFNa and IFNg commonly induced genes that bound any of the 4 components individually or in a random combination in their promoter region. (PDF 1359 KB) [file 18_2023_4830_MOESM4_ESM.pdf]

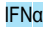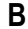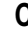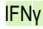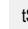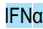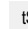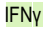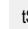

Supplement: Supplementary file 5 — Figure S5. Characterization of IFNα- and IFNγ-stimulated responses in WT, STAT1-, STAT2-, IRF9-, IRF1KO and IRF9/IRF1 dKO Huh7.5 cell lines. (A) WT, (B) STAT1-, (C) STAT2-, (D) IRF9-, (E) IRF1KO and (F) IRF9/IRF1 dKO Huh7.5 cells were treated with IFNα (1000 U/ml) or IFNγ (10 ng/ml) for the indicated time. The expression levels of STAT1, STAT2, IRF9 and IRF1 were evaluated by immunoblotting; p-phosphorylated, t- total. (PDF 2335 KB) [file 18_2023_4830_MOESM5_ESM.pdf]

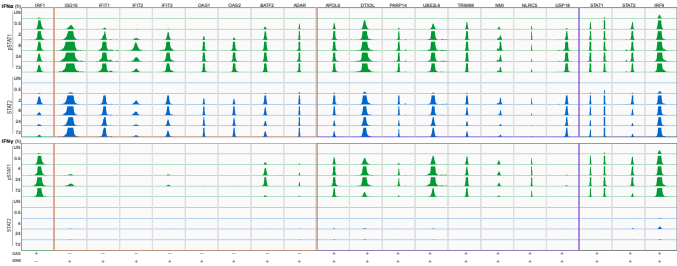

Supplement: Supplementary file 6 — Figure S6. pSTAT1 and STAT2 recruitment to GAS, ISRE and composite genes in IFNα and IFNγ treated Hu 7.5 cells. Views of the ChIP-seq peaks detected in the promoter regions of pre-selected GAS, ISRE and composite genes in untreated or IFNα and IFNγ treated Huh7.5 cells. All peaks were mapped onto human reference genome hg38 and visualized using the IGV genome browser; scale for pSTAT1 and STAT2 0-1000. (PDF 128 KB) [file 18_2023_4830_MOESM6_ESM.pdf]

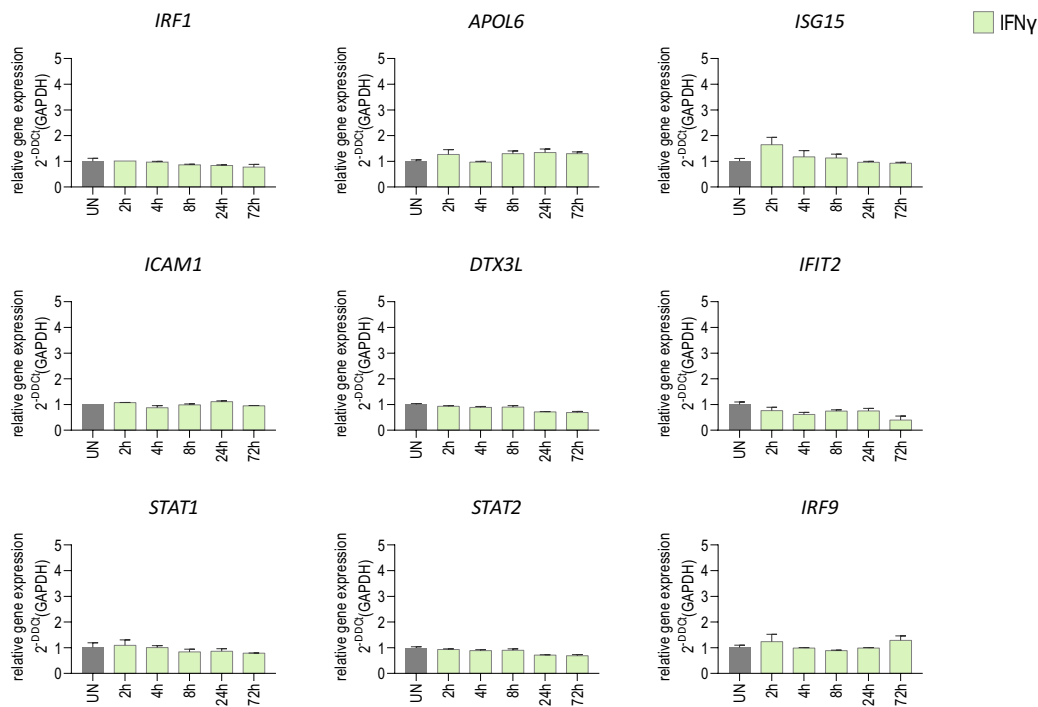

Supplement: Supplementary file 7 — Figure S7. Expression of GAS, ISRE and composite-containing representative genes in STAT1KO Huh7.5 cells after IFNγ treatment. GAS (IRF1, ICAM1), ISRE (ISG15, IFIT2) and composite (APOL6, DTX3L, STAT1, STAT2, IRF9) gene expression was analyzed using qPCR. Mean +/- SEM, n=2. (PDF 32 KB) [file 18_2023_4830_MOESM7_ESM.pdf]
