# Supplementary material for: Time-dependent recruitment of GAF, ISGF3 and IRF1 complexes shapes IFNα and IFNγ-activated transcriptional responses and explains mechanistic and functional overlap
Source: Cell Mol Life Sci. 2023 Jun 22;80(7):187. doi: 10.1007/s00018-023-04830-8 (PMC10287828; doi:10.1007/s00018-023-04830-8)
Supplement: Supplementary file 8 — Table S1. Primer sequences for RT-PCR and cloning. (DOCX 19 KB) [file 18_2023_4830_MOESM8_ESM.docx]

| qPCR_IRF1_F, AAAAGG AGCCAG ATCCCA AGA |
| --- |
| qPCR_IRF1_R, CATCCG GTACAC TCGCAC AG |
| qPCR_ICAM1_F, TGTGAC CAGCCC AAGTTG TT |
| qPCR_ICAM1_R, AGTCCA GTACAC GGTGAG GA |
| qPCR_MX1_F , ACCACA GAGGCT CTCAGC AT |
| qPCR_MX1_R, CTCAGC TGGTCC TGGATC TC |
| qPCR_OAS2_F, TCTGCC TCCCAT CCTACC ATT |
| qPCR_OAS2_R, CCATCT CGTCGA TCAGTG TCT |
| qPCR_APOL6_F, CGTCTT TCTCCA GCCCAG AC |
| qPCR_APOL6_R, CAAATG ATTTTC TTCTCT CCACGG |
| qPCR_PARP14_F, ACGATG AAATGA GGCGTT GTC |
| qPCR_PARP14_R, TGCCAG GTCTTG ATTCTC GG |
| qPCR_STAT1_F, TGTTAT GGGACC GCACCT TC |
| qPCR_STAT1_R, AGTGAA CTGGAC CCCTGT CT |
| qPCR_STAT2_F, TCGAAA CACCTG TGGAGA GC |
| qPCR_STAT2_R, GTCTTC CCTTTG GCCTGG AT |
| qPCR_IRF9_F, GAGCCA GACTAC TCACTG CTG |
| qPCR_IRF9_R, CCACTA GGATGC CCCTCT CA |
| qPCR_ISG15_F, GTGGAC AAATGC GACGAA CC |
| qPCR_ISG15_R, TCGAAG GTCAGC CAGAAC AG |
| qPCR_DTX3L_F, TTGTTG ACGAAA AACCTG TGC |
| qPCR_DTX3L_R, GTATGC CCTCTG CTCTTT GGA |
| qPCR_IFIT2_F, TCTTCC GTGTCT GTTCCA TTC |
| qPCR_IFIT2_R, AGCTGA AAGTTG CCATAC CG |
| qPCR_GAPDH_F, CAATAT GATTCC ACCCAT GGCAA |
| qPCR_GAPDH_R, CAATAT GATTCC ACCCAT GGCAA |
| qPCR_ACTB_F, ACAGAG CCTCGC CTTTGC CGAT |
| qPCR_ACTB_R, ATCATC CATGGT GAGCTG GCGG |
| ChIP_PCR_IRF1_F, CCAAAC ACTTAG CGGGAT TC |
| ChIP_PCR_IRF1_R, GAAATG ACGGCA CGCAG |
| ChIP_PCR_ICAM1_F, CACCCT GTCAGT CCGGAA AT |
| ChIP_PCR_ICAM1_R, GCGCGT GATCCT TTATAG CG |
| ChIP_PCR_IFIT2_F, AAACAT CCCTCT CTGCTG CC |
| ChIP_PCR_IFIT2_R, CAAGTG GCCTCT GGTTCC TT |
| ChIP_PCR_ISG15_F, CCGCCA CTTTTG CTTTTC CC |
| ChIP_PCR_ISG15_R, AGCACC GGCCCT ATTATA AGC |
| ChIP_PCR_STAT1prox_F, CGCTCA GCCAAT TAGACG C |
| ChIP_PCR_STAT1prox_R, GTAAAC AGAACG CCAGTT CCC |
| ChIP_PCR_STAT1dist_F, CTCTCA ATCCCA GTCCTT CTC |
| ChIP_PCR_STAT1dist_R, GAACCG CTTCGG AAACAG C |
| ChIP_PCR_STAT2_F, ACTTTC TACGAG GGGAGG AGT |
| ChIP_PCR_STAT2_R, AGGCGT CGAAAT TCCGGT C |
| ChIP_PCR_IRF9_F, AGATGC TGCTGC CCTCTA GT |
| ChIP_PCR_IRF9_R, CCCCTT TCTACA GTCCCC A |
| ChIP_PCR_APOL6_F, TCTCCT GCAGCA CTTAAC CG |
| ChIP_PCR_APOL6_R, CCTGAA CCTCTG TCCTTG CC |
| ChIP_PCR_UBE2L6_F, TTTCCC AAAGCC TGAGGA AGTT |
| ChIP_PCR_UBE2L6_R, ATTTTC TGTTCC TCTGAG TCAGGT |
| ChIP_PCR_TRIM69_F, TCTCAT CACTGC ATGGGG TG |
| ChIP_PCR_TRIM69_R, ACTGCC CAATCA AAACCC GA |
| ChIP_PCR_PARP14_F, CCTGGG TCCGCT GTGTTA G |
| ChIP_PCR_PARP14_R, CTCCGG GTAGAA GAACAC CAG |
| pXPG_IRF1_F, atctcgagctcggtacccATCAAGGTAGGGCTACTATT |
| pXPG_IRF1_R, tgccaagcttgtcgacccGGTCCACGCCGCGTC |
| pXPG_IRF1∆GAS_F, TGACGGACCCACCGTGACGGCACGCAGCCGGC |
| pXPG_IRF1∆GAS_R, ACGGTGGGTCCGTCAGGCTGTTGTAGAGCTAGCG |
| pXPG_ICAM1_F, atctcgagctcggtacccATGCCCGTGTCAGCTAGGT |
| pXPG_ICAM1_R, tgccaagcttgtcgacccCACCTCTCATCCCAGCTACTC |
| pXPG_ICAM1∆GAS_F, AGGTCCACGGCTTAGCAGCACCGCCCCTTGGCCC |
| pXPG_ICAM1∆GAS_R, CTAAGCCGTGGACCTCGCGCCTTCCCCTCCGG |
| pXPG_IFIT2_F, atctcgagctcggtacccGCTGAAAAGAACCCTTTTGT |
| pXPG_IFIT2_R, tgccaagcttgtcgacccACAGAGGTAGAGGCATAGTAAGG |
| pXPG_IFIT2∆ISRE_F, CTATCGCTACTGCTCCTTTTGTAACGTCAGCTGAAGGG |
| pXPG_IFIT2∆ISRE_R, GAGCAGTAGCGATAGAAAGTGAAATTGGCAGGACTC |
| pXPG_ISG15_F, atctcgagctcggtacccGGGCCTGGTGGTGCAC |
| pXPG_ISG15_R, tgccaagcttgtcgacccTCTCAGGGGTGACCTGCTTT |
| pXPG_ISG15∆ISRE1_F, TCGACGTTGTGCATTCCGCTCACTCTGGGGCATG |
| pXPG_ISG15∆ISRE1_R, AATGCACAACGTCGAATGACCGAAAGACAGGGAAAAGC |
| pXPG_ISG15∆ISRE2_F, GGGACATCTAGTCTGAAGCCAAATTTGGCCACCAGC |
| pXPG_ISG15∆ISRE2_R, CAGACTAGATGTCCCTTTCCCGAGGCATGCCC |
| pXPG_APOL6_F, atctcgagctcggtacccAGATGCTGTGGAGGAAAGCA |
| pXPG_APOL6_R, tgccaagcttgtcgacccCAGGTGCCTAAAGCCAACCC |
| pXPG_APOL6∆ISRE_F, AAGAGGACTGCCCGTGACAGCTGGAGCCCATGATTTC |
| pXPG_APOL6∆ISRE_R, ACGGGCAGTCCTCTTTTTGCCAGTTTCTCTAATTGGATG |
| pXPG_APOL6∆GAS_F , TGATGGACTGACCGAGCCCTAGAGCTTTGCTTTTTC |
| pXPG_APOL6∆GAS_R, TCGGTCAGTCCATCATGGGCTCCAGCTGTCAC |
| pXPG_APOL6∆ISRE∆GAS _F, TGATGGACTGACCGAGCCCTAGAGCTTTGCTTTTTC |
| pXPG_APOL6∆ISRE∆GAS _R, TCGGTCAGTCCATCATGGGCTCCAGCTGTCAC |
| pXPG_TRIM69_F, atctcgagctcggtacccTCTGGGCTCACTGCAACCTC |
| pXPG_TRIM69_R , tgccaagcttgtcgacccCTGGCTGCACTGGGCTG |
| pXPG_TRIM69∆GAS_F, CCCTACATCGACAAGCAGCTGGCTTTGAAGAGCTCG |
| pXPG_TRIM69∆GAS_R, CTTGTCGATGTAGGGGAAGGGCAGGGTTGCTTCC |
| pXPG_TRIM69∆ISRE_F , AAGAGCTCGAGCTCAGGACGGGAAAGTCCTGATTTC |
| pXPG_TRIM69∆ISRE_R , TGAGCTCGAGCTCTTCAAAGCCAGCTGCTTTCCG |
| pXPG_TRIM69∆ISRE∆GAS _F, CCCTACATCGACAAGCAGCTGGCTTTGAAGAGCTCG |
| pXPG_TRIM69∆ISRE∆GAS _R, CTTGTCGATGTAGGGGAAGGGCAGGGTTGC TTCC |
| pXPG_UBE2L6_F , atctcgagctcggtacccAGGAAGAGGGTGCCTGCTA |
| pXPG_UBE2L6_R , tgccaagcttgtcgacccCCCCCACCCCCAACATATAA |
| pXPG_UBE2L6∆GAS_F, TGATGCACTACACGGCAGTAGGAAAGAACTGTGTG |
| pXPG_UBE2L6∆GAS_R, CCGTGTAGTGCATCACCTGACTCAGAGGAACAG |
| pXPG_UBE2L6∆ISRE_F, CTAAGACCATACGCTGTTCCTCTGAGTCAGGTGATG |
| pXPG_UBE2L6∆ISRE_R, AGCGTATGGTCTTAGAAAGGCCATTAAGAGAGCC |
| pXPG_UBE2L6∆ISRE∆GAS _F, CTAAGACCATACGCTGTTCCTCTGAGTCAGGTGATG |
| pXPG_UBE2L6∆ISRE∆GAS _R, AGCGTATGGTCTTAGAAAGGCCATTAAGAGAGCC |
| pXPG_IRF9_F, atctcgagctcggtacccGTCCCAGGCCTGACTCCA |
| pXPG_IRF9_R, tgccaagcttgtcgacccTCAGTCTAACAGAGCGACCC |
| pXPG_IRF9∆ISRE_F , AGGCAGGCTGCCGATTAGGGTGGGGACTGTAGAAAGGG |
| pXPG_IRF9∆ISRE_R, ATCGGCAGCCTGCCTCCAGGGAGGGCCTTTTCC |
| pXPG_IRF9∆GAS_F, TGACGGATGGACCGAGGCCCTCCCTGGAGGAG |
| pXPG_IRF9∆GAS_R, TCGGTCCATCCGTCACGTGGTCTGAGTTGCAGG |
| pXPG_IRF9∆ISRE∆GAS _F, AGGCAGGCTGCCGATTAGGGTGGGGACTGTAGAAAGGG |
| pXPG_IRF9∆ISRE∆GAS _R, ATCGGCAGCCTGCCTCCAGGGAGGGCCTTCG |
| pXPG_STAT2_F, atctcgagctcggtacccAATTATCCTGGCGGAGTTATGCA |
| pXPG_STAT2_R, tgccaagcttgtcgacccCGCGACTTCCCGTCCCTAG |
| pXPG_STAT2∆ISRE_F , CTAAGCGCGGCCACGGGGCTCACGCGCGGGTTCTCG |
| pXPG_STAT2∆ISRE_R, CGTGGCCGCGCTTAGCTTGAGGAGCCTGGAGACG |
| pXPG_STAT2∆GAS_F , CGGGGCGCGACGGATCAGCTGTTTCAGTCTTGGGC |
| pXPG_STAT2∆GAS_R, ATCCGTCGCGCCCCGCGCGTGAGCCCCGG |
| pXPG_STAT2∆ISRE∆GAS _F , CGGGGCGCGACGGATCAGCTGTTTCAGTCTTGGGC |
| pXPG_STAT2∆ISRE∆GAS _R, ATCCGTCGCGCCCCGCGCGTGAGCCCCGTGG |
| pXPG_STAT1prox_F , atctcgagctcggtacccCCATGCTTCCGAGCTGTCAA |
| pXPG_STAT1prox_R, tgccaagcttgtcgacccCTGCTCGCACTTGGAATACTC |
| pXPG_STAT1dist_F, atctcgagctcggtacccTCTGAGTCAGTGCAAGTGCA |
| pXPG_STAT1dist_R, tgccaagcttgtcgacccGAATGATGTTCATAGCAGCTTCA |
| pXPG_STAT1dist/prox_Fp, acatcattcCCATGCTTCCGAGCTGTCAA |
| pXPG_STAT1dist/prox_Rp, tgccaagcttgtcgacccCTGCTCGCACTTGGAATACTC |
| pXPG_STAT1dist/prox_Fd, atctcgagctcggtacccTCTGAGTCAGTGCAAGTGCA |
| pXPG_STAT1dist/prox_Rd, gaagcatggGAATGATGTTCATAGCAGCTTCA |
| pXPG_STAT1prox∆ISRE_F , GTAAGCGCGCGCGCCTGCGCAGAGTCTGCGGAGG |
| pXPG_STAT1prox∆ISRE_R, GGCGCGCGCGCTTACCCGGCAGGAGAAAAGGC |
| pXPG_STAT1dist_∆ISRE_F, TAAAGCGAGCGGCTCGGTTTCCTCTCAATCCCAGTCC |
| pXPG_STAT1dist_∆ISRE_R, GAGCCGCTCGCTTTAGAGCCTGCGGGAGCAGTACG |
| pXPG_STAT1dist_∆GAS_F, CAGAGCTCGAGCTCGCACGTGGGGCGGCTCTTC |
| pXPG_STAT1dist_∆GAS_R , CGAGCTCGAGCTCTGCCCGGGAGCGAGAAGG |
| pXPG_NMI_F, ATCTCG AGCTCG GTACCC ACAAGC TCCACA ACAAAA CAAGA |
| pXPG_NMI_R, TGCCAA GCTTGT CGACCC AGCTTG CAGTGA GCCGAG AT |
| pXPG_NMI_∆ISRE_F, TCACTCGTGCTCTTAGTTTTTTTTTCTGTTAGTGACT |
| pXPG_NMI_∆ISRE_R, TAAGAGCACGAGTGATTTTTAAAAAGGGGTGGTTTTGC |
| pXPG_NMI_∆GAS_F, TGTTACACGGACAGGGCAGGCGCGCTGGGCCTTGG |
| pXPG_NMI_∆GAS_R, CCTGTCCGTGTAACAGCAGCGCCTGAAACGCC |
| pXPG_PARP14_F, atctcgagctcggtacccCCACAAAAAAAACTGTGAGTGT |
| pXPG_PARP14_R, tgccaagcttgtcgacccGCCCCGGGTGCGC |
| pXPG_PARP14_∆ISRE_F, ACACTCGCGCTCGAGTCAAAGTTAGCGGCCCGG |
| pXPG_PARP14_∆ISRE_R, CTCGAGCGCGAGTGTTTCCTGGAAAACTCCCAGGC |
| pXPG_PARP14_∆GAS_F, GTTACACAGACAACGAAAGCGAAAGAGTCAAAGTTAGC |
| pXPG_PARP14_∆GAS_R, CGTTGTCTGTGTAACTCCCAGGCCTTGGTTTCC |
| pXPG_PARP14_∆GAS(Pmut)_F, GTTACACAGGAAACGAAAGCGAAAGAGTCAAAGTTAGC |
| pXPG_PARP14_∆GAS(Pmut)_R, CGTTTCCTGTGTAACTCCCAGGCCTTGGTTTCC |
| pXPG_PARP14_∆ISRE(Pmut)_F, ACGAAAGCGCTCGAGTCAAAGTTAGCGGCCCGG |
| pXPG_PARP14_∆ISRE(Pmut)_R, CTCGAGCGCTTTCGTTTCCTGGAAAACTCCCAGGC |
