# Supplementary material for: Time-dependent recruitment of GAF, ISGF3 and IRF1 complexes shapes IFNα and IFNγ-activated transcriptional responses and explains mechanistic and functional overlap
Source: Cell Mol Life Sci. 2023 Jun 22;80(7):187. doi: 10.1007/s00018-023-04830-8 (PMC10287828; doi:10.1007/s00018-023-04830-8)
Supplement: Supplementary file 9 — Table S2. List of IFNα- and IFNγ-specific integrated genes. (DOCX 24 KB) [file 18_2023_4830_MOESM9_ESM.docx]

| IFNα-specific | | | | | | IFNγ-specific | | | | | |
| --- | --- | --- | --- | --- | --- | --- | --- | --- | --- | --- | --- |
| gene | motif | gene | motif | gene | motif | gene | motif | gene | motif | gene | motif |
| AASS | GAS | AKAP7 | IISRE | BAZ2A | composite | ABCB1 | GAS | BCL2L13 | ISRE | APOL3 | composite |
| AMOTL2 |  | ANKFY1 |  | BISPR |  | ASGR1 |  | CIR1 |  | CARD16 |  |
| ANAPC4 |  | APH1A |  | BTC |  | ATAD5 |  | DUSP10 |  | CXCL11 |  |
| AUP1 |  | AZI2 |  | CAVIN2 |  | BCL6 |  | IL15RA |  | ENPEP |  |
| B3GALNT1 |  | BAHCC1 |  | CHMP5 |  | C1orf198 |  | MAL2 |  | FAS |  |
| B3GAT3 |  | C4orf33 |  | CXCL10 |  | C4BPB |  | MEIS1 |  | IL18BP |  |
| CCDC9 |  | CD164 |  | CYBC1 |  | CAND2 |  | NPTX2 |  | KDSR |  |
| CDC7 |  | CDK18 |  | CYP2J2 |  | CD63 |  | NR3C1 |  | MMAA |  |
| CNTNAP1 |  | CMTR1 |  | DCLRE1C |  | CDIPT |  | OXSR1 |  | UTRN |  |
| DEPP1 |  | DUSP16 |  | EDEM2 |  | CFH |  | PKIB |  |  |  |
| DNAJB1 |  | ECHS1 |  | EPSTI1 |  | CFL2 |  | PPA1 |  |  |  |
| DNAJB9 |  | FAM111A-DT |  | FMR1 |  | COQ8B |  | PSMA3 |  |  |  |
| GPR37 |  | FYCO1 |  | GPR180 |  | CRKL |  | PSMA4 |  |  |  |
| GTPBP2 |  | GRWD1 |  | HERC5 |  | CYP1B1 |  | PSMA6 |  |  |  |
| HDAC7 |  | HAX1 |  | IFI27 |  | DDX23 |  | PSMB2 |  |  |  |
| HP |  | HNRNPD |  | IFI44 |  | FKBP4 |  | TXN |  |  |  |
| HPR |  | IFI44L |  | IFIH1 |  | GAS8 |  | XPO6 |  |  |  |
| IRAK4 |  | KMO |  | IFITM1 |  | GBP5 |  |  |  |  |  |
| LPAR6 |  | LTBR |  | KBTBD2 |  | GCLM |  |  |  |  |  |
| LRG1 |  | MX2 |  | LAMP3 |  | GSTK1 |  |  |  |  |  |
| MIIP |  | NADK |  | LRP10 |  | HAUS3 |  |  |  |  |  |
| NRSN2 |  | NT5C3A |  | RANGAP1 |  | HEATR1 |  |  |  |  |  |
| OSGIN2 |  | NUP160 |  | RASGRP3 |  | HELB |  |  |  |  |  |
| PGM3 |  | OASL |  | RFC2 |  | HSD3B7 |  |  |  |  |  |
| PLCE1 |  | PLEKHA4 |  | RSAD2 |  | KIF24 |  |  |  |  |  |
| PNPLA8 |  | PPP2R2A |  | SRRM1 |  | KIF2A |  |  |  |  |  |
| PSMD3 |  | PSAPL1 |  | TRIM22 |  | LEPROTL1 |  |  |  |  |  |
| RAB1B |  | RAD9A |  | TRIM5 |  | LIMA1 |  |  |  |  |  |
| SEMA4B |  | RBBP6 |  | TRIP12 |  | LMO7 |  |  |  |  |  |
| SERPINA10 |  | RRAS |  | TXNL4B |  | LRRC61 |  |  |  |  |  |
| SLC9B2 |  | SCARB2 |  | ZNF107 |  | NEK8 |  |  |  |  |  |
| SNAPIN |  | SNX3 |  |  |  | NEURL3 |  |  |  |  |  |
| SVIP |  | SNX6 |  |  |  | NT5C2 |  |  |  |  |  |
| TEAD4 |  | STARD4 |  |  |  | PNISR |  |  |  |  |  |
| TSPAN4 |  | STX7 |  |  |  | PSPH |  |  |  |  |  |
| TTBK2 |  | TCF7L2 |  |  |  | RBM3 |  |  |  |  |  |
| ZBTB42 |  | TMEM62 |  |  |  | RNF115 |  |  |  |  |  |
|  |  | TNFSF10 |  |  |  | SNTB2 |  |  |  |  |  |
|  |  | ZFP36L1 |  |  |  | SSBP4 |  |  |  |  |  |
|  |  | ZNF620 |  |  |  | STEAP4 |  |  |  |  |  |
|  |  |  |  |  |  | SULT1C2 |  |  |  |  |  |
|  |  |  |  |  |  | TCERG1 |  |  |  |  |  |
|  |  |  |  |  |  | TGIF1 |  |  |  |  |  |
|  |  |  |  |  |  | TLCD2 |  |  |  |  |  |
|  |  |  |  |  |  | TMEM126B |  |  |  |  |  |
|  |  |  |  |  |  | TP53BP1 |  |  |  |  |  |
|  |  |  |  |  |  | WDR25 |  |  |  |  |  |
|  |  |  |  |  |  | YWHAG |  |  |  |  |  |
|  |  |  |  |  |  | ZNF670 |  |  |  |  |  |
