# Supplementary material for: Time-dependent recruitment of GAF, ISGF3 and IRF1 complexes shapes IFNα and IFNγ-activated transcriptional responses and explains mechanistic and functional overlap
Source: Cell Mol Life Sci. 2023 Jun 22;80(7):187. doi: 10.1007/s00018-023-04830-8 (PMC10287828; doi:10.1007/s00018-023-04830-8)
Supplement: Supplementary file 10 — Table S3. List of IFNα- and IFNγ-common integrated GAS, ISRE and composite genes. (DOCX 19 KB) [file 18_2023_4830_MOESM10_ESM.docx]

| common genes | | | | | | | |
| --- | --- | --- | --- | --- | --- | --- | --- |
| gene | | motif | gene | | motif | gene | motif |
| A2M | LSR | GAS | ACOT7 | NOD1 | ISRE | AIG1 | composite |
| AGFG2 | MAFF |  | ACSL5 | OAS1 |  | APOL1 |  |
| AGT | MAN1A1 |  | ACY3 | OAS2 |  | APOL2 |  |
| ANXA2R | MAPKAPK3 |  | ADAR | OAS3 |  | APOL6 |  |
| APOA2 | MED16 |  | AIDA | OGFR |  | CASP1 |  |
| ASAP1 | MITD1 |  | AP5Z1 | OPTN |  | CASP4 |  |
| ASXL1 | MTPN |  | B2M | OTUD4 |  | CD274 |  |
| BCL6 | NAMPT |  | BATF2 | PANX1 |  | CFB |  |
| BCLAF1 | NFKB2 |  | BTN3A1 | PARP10 |  | CSF1 |  |
| BOD1 | NFKBIZ |  | BTN3A2 | PCNA |  | CTSO |  |
| BTBD1 | NNMT |  | BTN3A3 | PNPT1 |  | DDX58 |  |
| C19orf12 | NUCB1 |  | C5orf15 | PRKD2 |  | DTX3L |  |
| C1RL | OSMR |  | CASP7 | PRRG4 |  | GBP3 |  |
| C1S | PDE4D |  | CASP8 | PSMB10 |  | IFI35 |  |
| C2 | PLEKHB2 |  | CCND1 | PSME1 |  | IFITM3 |  |
| C4BPB | PROS1 |  | CEACAM1 | RBCK1 |  | IRF2 |  |
| C5 | PSMB3 |  | CMPK2 | RBM43 |  | IRF9 |  |
| CREBRF | RAB27A |  | CYTH1 | RPS12 |  | LGALS3BP |  |
| CUTA | RAD1 |  | DDX60 | RTP4 |  | MDK |  |
| DDX23 | RAP1A |  | DDX60L | SAMD9 |  | MVB12A |  |
| DEK | RBM42 |  | DNAJA1 | SEC16B |  | MX1 |  |
| DPAGT1 | RBP4 |  | DNPEP | SLC15A3 |  | MYD88 |  |
| EFNA1 | SCN9A |  | ERAP1 | SLFN12 |  | NCOA7 |  |
| FANCI | SDSL |  | FAM111A | SMARCA5 |  | NMI |  |
| FGA | SEPHS2 |  | GBP1 | SOCS1 |  | PARP14 |  |
| FGB | SERPINB1 |  | GBP1P1 | SP100 |  | PHF11 |  |
| FGL1 | SERPING1 |  | GNA13 | SP140L |  | PLSCR1 |  |
| GADD45B | SHC2 |  | GNB4 | STAT1 |  | PML |  |
| GCLM | SKI |  | GSDMD | STAT2 |  | PRRC2C |  |
| GCNT3 | SLC25A18 |  | IFI6 | TAP1 |  | RBM7 |  |
| GEN1 | SLC7A2 |  | IFIT1 | TAP2 |  | RIPK1 |  |
| GNB2 | SMPD1 |  | IFIT2 | TAPBP |  | RNF213 |  |
| H6PD | SOCS3 |  | IFIT3 | TAPBPL |  | RPS6KB2 |  |
| HABP2 | SSBP4 |  | IFIT5 | TMEM140 |  | SERPIND1 |  |
| HIPK3 | STAT3 |  | ISG15 | TRIM14 |  | SHISA5 |  |
| HLA-E | TCIRG1 |  | ISG20 | TRIM26 |  | SMG1 |  |
| HNRNPR | TNFAIP1 |  | KDSR | TRIM44 |  | SP110 |  |
| HPX | TOP1 |  | LAP3 | USF1 |  | SPTBN1 |  |
| ICAM1 | UBE2D1 |  | MOV10 | XAF1 |  | STX17 |  |
| IMPAD1 | VTN |  | NAPA | ZC3H7B |  | TRIM69 |  |
| INTS10 | WSB1 |  | NEDD1 | ZFYVE26 |  | UBE2L6 |  |
| IPO8 | YPEL2 |  |  |  |  | USP18 |  |
| IRF1 | ZNF24 |  |  |  |  | ZC3HAV1 |  |
