# Supplementary material for: Time-dependent recruitment of GAF, ISGF3 and IRF1 complexes shapes IFNα and IFNγ-activated transcriptional responses and explains mechanistic and functional overlap
Source: Cell Mol Life Sci. 2023 Jun 22;80(7):187. doi: 10.1007/s00018-023-04830-8 (PMC10287828; doi:10.1007/s00018-023-04830-8)
Supplement: Supplementary file 11 — Table S4. List of IFNα- and IFNγ-common integrated composite genes with spacing between and orientation of GAS and ISRE sites. (DOCX 19 KB) [file 18_2023_4830_MOESM11_ESM.docx]

| No. | gene | ISRE | linker | GAS | orientation |
| --- | --- | --- | --- | --- | --- |
| consensus | | AG-TTT-CNN-TTT-CN |  | -TTC-CNG-GAA- |  |
| 1 | IFI35 | AC-TTT-CA--TTT-CC | overlap | TTTC-CGT-GAAA | - |
| 2 | MYD88 | GC-TTT-CGC-TTT-CC | overlap | -TTC-TCG-GAAA | - |
| **3** | **PARP14** | **TC-TTT-CGC-TTT-CG**  **TC-TTT-CGC-TTT--CG-TTTCC** | **overlap**  **0nt** | **TTTC-CTG-GAAA** | **-** |
| 4 | IFITM3 | AG-TTT-CCT-TTT-CC  AG-TTT-CGG-TTT-CT | overlap  12nt | TTTC-CTG-GCAT | - |
| 5 | LGALS3BP | AC-TTT-CGA-TTT-CC | overlap  23nt | TTTC-CAG-AAAC  TTC-CGG-GAT | - |
| 6 | MVB12A | AG-TTT-CAG-TTT-CC | overlap  54nt | TTTC-CCA-GAAA  -TTC-CAG-GAAG | - |
| 7 | SP110 | AC-TTT-CAC-TTT-TC | 1nt | TTTC-TCG-GAA | + |
| 8 | IRF2 | AA-TTT-CAT-TTT-CG | 3nt | TTTC-TCG-GAAA | + |
| 9 | APOL2 | AC-TTT-CAC-TTT-CC  AC-TTT-CCC-TTT-CG | 5nt  11nt | TTC-CCA-GCAG | - |
| 10 | CD274 | GC-TTT-CAG-TTT-AG | 6nt | TTTC-ACC-GAA | + |
| 11 | GBP3 | AC-TTT-CAG-TTT-CA | 6nt | TTTC-AAG-GAAG | + |
| 12 | APOL1 | AC-TTT-CAC-TTT-CC  AC-TTT-CCC-TTT-CG | 6nt  12nt | -CTG-CTG-GGAA | - |
| 13 | STX17 | GG-TTT-CGT-TTT-TT | 7nt | -TTA-TTG-GAA | + |
| 14 | SHISA5 | AG-TTT-CAA-TTT-CC | 10nt | TTTT-GGA-GAAA | - |
| **15** | **TRIM69** | **GG-TTT-CTC-TTT-CT** | **14nt** | **TTTC-CGA-GAAA** | **-** |
| 16 | MX1 | AG-TTT-CGG-TTT-CA | 15nt | -TTT-CTG-GAAA | - |
| 17 | PARP9 | AG-TTT-CAG-TTT-CG | 17nt | -TTC-CCT-GGA | + |
| **18** | **DTX3L** | **AG-TTT-CAG-TTT-CG** | **17nt** | **-TTC-CCT-GGA** | **-** |
| 19 | PLSCR1 | GG-TTT-CCT-TTT-CC | 17nt | TTTC-CT--GAA | + |
| 20 | PHF11 | GG-TTT-CGT-TTT-CT | 17nt | -TTC-CGG-GAT | + |
| **21** | **APOL6** | **AC-TTT-CAG-TTT-CC** | **18nt** | **TTTC-CTG-GAA** | + |
| **22** | **UBE2L6** | **AC-TTT-CAT-TTT-CT** | **19nt** | **-TAC-TAG-GAAA** | + |
| 23 | RPS6KB2 | AG-TTT-CAT-TTT-CT | 32nt | -TTC-CAG-GAAA | + |
| 24 | USP18 | AG-TTT-CGC-TTT-CC | 37nt | -TTC-CCC-CGC | - |
| 25 | RIPK1 | AC-TTT-GC--TTT-CC | 42nt | -TTC-CCG-GAA | + |
| 26 | CFB | AG-TTT-CTG-TTT-CC | 46nt | TTC-CGG-GAAA | + |
| 27 | NCOA7 | AG-TTT-CTG-TTT-CC | 49nt | -TTT-GCT-CTAA | - |
| 28 | CASP1 | AC-TTT-CAG-TTT-CA | 51nt | TTC-TTG-GAAC | + |
| 29 | CSF1 | AC-TTT-CAG-TTT-CC | 66nt | TTTC-CCA-TAAA | - |
| 30 | CASP4 | AC-TTT-CAT-TTT-TG | 77nt | -TTA-TCAG-GAA | - |
| 31 | MDK | TC-TTT-CAC-TTT-CA | 92nt | TTTG-GGG-GAAC | + |
| 32 | RBM7 | CC-TTT-CGT-TTT-CC | 124nt | TTTC-CTG-GAA | + |
| 33 | SERPIND1 | GG-TTT-CAT-TTT-TC | 134nt | -TTC-TCA-GAAA | - |
| 34 | SPTBN1 | AG-TTT-CGG-TTC-CC | 144nt | -TTC-CAG-GAAA | + |
| 35 | SMG1 | AT-TTT-CAC-TTT-CC | 145nt | -TTC-CCG-GAAA | - |
| 36 | CTSO | TC-TTT-CGG-TTC-CT | 169nt | -TAC-CCG-GAAG | + |
| 37 | PRRC2C | AG-TTT-TAC-TTT-CA | 169nt | -TTC-TTA-GAAA | + |
| 38 | AIG1 | AC-TTT-CTC-TTT-AC | 188nt | TTTA-CTG-GAAG | + |
| 39 | PML | AG-TTT-CGA-TTC-TC | 233nt | TTTA-CCG-TAA | - |
| 40 | DDX58 | AG-TTT-CG--TTT-CC | 254nt | -ATC-CTG-GAAG | + |
| **41** | RNF213 | CC-TTT-CCA-TTT-GG  GA-TTT-CAC-TTT-CG | 259nt  273nt | -TTC-CAG-GAAA | + |
| 42 | **NMI** | **AA-TTT-CAC-TTT-CG** | **274nt** | **-TTC-CCG-GAAA** | + |
| 43 | ZC3HAV1 | AG-TTT-CGA-TTT-CC | 415nt | -TTC-CCG-GAAA | - |
